# Early Prediction of Alzheimer’s Disease Using Null Longitudinal Model-Based Classifiers

**DOI:** 10.1371/journal.pone.0168011

**Published:** 2017-01-03

**Authors:** Giovana Gavidia-Bovadilla, Samir Kanaan-Izquierdo, María Mataró-Serrat, Alexandre Perera-Lluna, for the Alzheimer’s Disease Neuroimaging Initiative

**Affiliations:** 1 Department of ESAII, Universitat Politècnica de Catalunya, Barcelona, Catalonia, Spain; 2 Department of ESAII, Center for Biomedical Engineering Research (CREB), Universitat Politècnica de Catalunya, Barcelona, Catalonia, Spain; 3 Department of Clinical Psychology and Psychobiology, Universitat de Barcelona, Barcelona, Catalonia, Spain; 4 Institute of Neurosciences, Universitat de Barcelona, Barcelona, Catalonia, Spain; 5 CIBER of Bioengineering, Biomaterials and Nanomedicine (CIBER-BBN), Barcelona, Catalonia, Spain; Banner Alzheimer’s Institute, UNITED STATES

## Abstract

Incipient Alzheimer’s Disease (AD) is characterized by a slow onset of clinical symptoms, with pathological brain changes starting several years earlier. Consequently, it is necessary to first understand and differentiate age-related changes in brain regions in the absence of disease, and then to support early and accurate AD diagnosis. However, there is poor understanding of the initial stage of AD; seemingly healthy elderly brains lose matter in regions related to AD, but similar changes can also be found in non-demented subjects having mild cognitive impairment (MCI). By using a Linear Mixed Effects approach, we modelled the change of 166 Magnetic Resonance Imaging (MRI)-based biomarkers available at a 5-year follow up on healthy elderly control (HC, n = 46) subjects. We hypothesized that, by identifying their significant variant (*vr*) and quasi-variant (*qvr*) brain regions over time, it would be possible to obtain an age-based null model, which would characterize their normal atrophy and growth patterns as well as the correlation between these two regions. By using the null model on those subjects who had been clinically diagnosed as HC (n = 161), MCI (n = 209) and AD (n = 331), normal age-related changes were estimated and deviation scores (residuals) from the observed MRI-based biomarkers were computed. Subject classification, as well as the early prediction of conversion to MCI and AD, were addressed through residual-based Support Vector Machines (SVM) modelling. We found reductions in most cortical volumes and thicknesses (with evident gender differences) as well as in sub-cortical regions, including greater atrophy in the hippocampus. The average accuracies (ACC) recorded for men and women were: AD-HC: 94.11%, MCI-HC: 83.77% and MCI converted to AD (cAD)-MCI non-converter (sMCI): 76.72%. Likewise, as compared to standard clinical diagnosis methods, SVM classifiers predicted the conversion of cAD to be 1.9 years earlier for females (ACC:72.5%) and 1.4 years earlier for males (ACC:69.0%).

## Introduction

AD is a disease with both brain pathological processes and clinical decline occurring gradually, with dementia representing the last stage of many years of accumulation of these pathological changes. Changes begin several years before the onset of clinical symptoms and there is strong supporting evidence that it is related to the early abnormal production of *β*−amyloid (A*β*) peptide in the Cerebrospinal fluid (CSF). *β*− amyloidosis and the increase of CSF- *τ* protein precede neuronal dysfunction and neurodegeneration; and all of these precede cognitive changes and correlate with clinical symptom severity [1, 2]. Neurodegeneration begins with a typical pattern of early neurofibrillary tangles in medial temporal lobe structures, mainly entorhinal cortex and hippocampus, and subsequently extending throughout most of the temporal lobe and the posterior cingulate. Finally, this pattern involves extensive cortical regions, especially parietal, prefrontal and orbitofrontal; and in an advance stage, change in several brain structures correlate closely with changes in cognitive [3, 4]. In addition to the foregoing, it is known that the apolipoprotein E (APOE) *ϵ*4 allele is the most prevalent genetic risk factor for AD [5]. Carriers of *ϵ*4 attain AD at an earlier age than non-carriers [1, 5]; this may be due to *ϵ*4 being involved in early A*β* plaque concentration and neuro-degeneration. However, in spite of these findings, it is important to be clear that *ϵ*4 carriers merely inherit an increased risk of developing the disease, but not all people with AD are *ϵ*4 carriers, and not all *ϵ*4 carriers will develop the disease.

Indeed, to understand the specific effects of AD on brain structures, it is important to study and differentiate their age-related changes in the absence of disease. Ageing causes changes at all levels, from molecular to morphological, in the brain which include shrinking. Studies have demonstrated that seemingly healthy subjects lose brain matter over time and brain age-related changes and function are not uniform across the whole brain or over subjects. The volume of the brain decreases with age at a rate of approximately 0.2–0.5% per year [6] and this rate might be even greater over the age of 70 [7]. Regionally, ageing-related atrophy has been observed across many of the cortical regions [6, 8, 9] with a prominent decline in the prefrontal cortex and the slight decline of the temporal cortex and parahippocampal cortex [8]. Decline has been also found in several subcortical structures including the caudate nucleus, amygdala, cerebellum and hippocampus [6, 9], the latter being the most studied structure, with annual atrophy rate of about 2.0% [6]. Studies on cross-sectional and longitudinal MRI have found significant correlations between gender and cortical and subcortical regions, but there are inconsistencies between the results [8, 9]. Finally, another factor to consider with regard to the effects of ageing on the brain and cognition is the influence of socio-demographic characteristics. Both higher levels of education or occupational attainment may act as a protective factor. For example, studies have found that higher levels of education may increase the brain reserve, leading to larger brain structures, which can help to counter the effects of brain atrophy [9, 10].

However, many of these age-related changes are shared by neurodegenerative diseases. Some studies have found that cognitively normal elderly (HC) have A*β* deposition in the brain with similar levels of the substances observed in subjects with dementia due to AD [11]. Similarly, despite an increase in *τ* protein having been seen in AD as compared to HC, *τ* deposition is found in other neurodegenerative diseases referred to as tauopathies [12]. At the macroscopic level, ageing has an effect on brain morphology with atrophy caused by dendritic pruning and loss of synapses and neurons. Part of this atrophy occurs in areas vulnerable to AD, while other changes have been observed in areas less characteristic of early-stage AD [6, 8, 9]. Because of the shared biochemical and morphological characteristics, it must be pointed out that it is a complex task to discriminate some of these age-related changes in healthy elderly subjects from subjects affected with early stage of AD.

In clinical practice, neuropsychological tests such as, the Clinical Dementia Rating (CDR) [13], the Mini-Mental Examination Score (MMSE) [14] and the Alzheimer’s Disease Assessment Scale cognitive subscale are used to monitor AD progression or treatment efficacy. However, although these tests unquestionably reflect an important aspect of disease progression (i.e. functional impairment), they also have several limitations such as relatively low specificity [15] and reliability [16]. During the past few years, there has been a considerable effort to identify additional biomarkers as early indicators of pathological changes to improve the accuracy of the clinical diagnosis of possible/probable AD and the prediction of disease progression from Mild Cognitive Impairment (MCI) which is a symptomatic predementia phase of AD [17]. In spite of CSF-A*β* and CSF-*τ* have been suggested as the most informative AD biomarkers [18]; several studies also suggested their combination with other features types, such as Magnetic Resonance Imaging (MRI)-based biomarkers, to increase early prediction accuracy [19]. Currently, neuroimaging and specifically structural MRI biomarkers support earlier and more precise diagnosis and measurement of progression. MRI Biomarkers of atrophy of medial temporal regions have been validated for early diagnosis of subjects at the MCI stage. Furthermore, MRI images used to measure rates of whole-brain and hippocampal region are considered powerful markers of progression of neurodegeneration being used as outcomes in clinical trials [4].

Alternatively, studies based on high dimensional methods have approached the HC/MCI/AD early prediction problem of automatically classifying brain regions and subjects from structural MRI-based biomarkers. These biomarkers allow the careful examination of morphological changes and their correlation with the progression of cognitive impairment. Multivariate techniques such as Orthogonal Partial Least Squares (OPLS), Support Vector Machine (SVM) and Relevance Vector Machine (RVM), among others, have enabled the correlation and variance of these biomarkers to be evaluated in a more easily interpreted way and with greater statistical power as compared to univariate approaches. Likewise, MRI biomarkers have been used in combination with other types of biomarkers, such as Positron Emission Tomography (PET)-based features, CSF-based biomarkers, Genetic-based biomarkers and socio-demographic and neuropsychological features to improve early diagnosis performance. Recent studies have proposed an age estimation framework by training RVM for regression (RVR) from MRI-based biomarkers of healthy subjects, which was used to estimate the age of MCI and AD subjects and to recognize faster brain atrophy [20, 21] and to predict the conversion from MCI to AD [22]. Other studies have proposed methods to calculate an MRI-based AD severity index from cross-sectional [23] and longitudinal studies [24], where multivatiate models based on OPLSR were built from HC and AD subject data. Models were applied to MCI subjects for early prediction of conversion to AD through subject classification as either healthy control-like or as AD-like. Besides the above-mentioned methods, studies have also applied SVM algorithms to improve prediction quality by selecting the most significant brain structures or other feature types in HC, MCI and AD subjects [3, 19, 25–29]; and to discriminate between these subject groups [3, 19, 25–28, 30, 31]. However, in spite of applied multivariate techniques and the features used, all these studies are focused mainly on three classification experiments: (1) HC vs AD, (2) HC vs MCI and (3) MCI vs AD; where the last one is mainly directed at discriminating between the stable MCI (sMCI, MCI who had not converted to AD) vs converted to AD (cAD, subjects who had converted to AD). For HC vs AD, the methods mentioned achieved high accuracy values (up to 94.9% sensitivity and 96.33% specificity); and from 78% to 85% on MCI vs AD. However, for the detection of prodromal AD (sMCI vs cAD), the sensitivity is substantially lower (below 60%) for most methods.

In this study, we aimed to characterize the age-related changes in brain structures and to identify the variant (*vr*) and quasi-variant (*qvr*) MRI biomarkers to build ageing-based null models. Null models were built from identified HC subjects (n = 46) with a normal CSF-profile. Most importantly, we addressed the HC/MCI/AD subject classification and the early prediction of conversion to AD, by using these null models to estimate the age-related values of *vr* and *qvr* MRI biomarkers for longitudinal data of HC (n = 161), MCI (n = 209) and AD (n = 331) subjects. Residuals were then calculated as deviation scores of observed MRI-based biomarkers from estimated normal MRI-based biomarkers. Support vector machines (SVM) were used to build residual-based classifiers for three experiments: MCI vs HC, AD vs MCI and AD vs HC. The advancement for early disease prediction was calculated as the number of years that the proposed method leads in predicting the last known subject diagnostic. Data used in this study was obtained from the Alzheimer’s Disease Neuroimaging Initiative (ADNI) study (adni.loni.usc.edu).

## Data

### ADNI cohorts

The ADNI was launched in 2003 as a public-private partnership, led by Principal Investigator Michael W. Weiner, MD. The primary goal of ADNI has been to test whether serial MRI, PET, other biological markers, and clinical and neuropsychological assessment can be combined to measure the progression of MCI and AD. For up-to-date information, see www.adni-info.org.

The complete ADNI project enrolled 819 adult subjects, aged 55—90 years, and recruited from over 50 sites across the United States and Canada. These subjects met entry criteria for a clinical diagnostic at baseline (see S1 Appendix) of late MCI (LMCI, n = 398), early probable AD (n = 193) and control normal (CN, n = 229) [32]. ADNI has been used by many number of publications focused on the characterization of age-related brain changes [6, 33] and the early prediction of conversion to AD [19, 23, 31, 34, 35]; a recent review has been published by Weiner and colleagues in Alzheimer & Dementia journal [36]. Details about the procedures for selection of participants and the full study protocol have been presented in [16, 37]. Also, details of the acquisition of structural MRIs of the participants can be found in *ADNI project site* (adni.loni.usc.edu).

#### CSF biomarkers

The ADNI CSF-based biomarkers were measured for A*β* 1 to 42 peptide (CSF-A*β*), total tau (CSF-*τ*) and *τ* phosphorylated at the theorine 181 P-*τ*_181*P*_ concentrations using the xMAP platform (Luminex Corp, Austin, Texas) and INNO-BIA AlzBio3 research-use-only reagents. Biomarkers analysis applied by ADNI are described in detail at http://adni.loni.usc.edu/methods/biomarker-analysis/. CSF biomarkers set was conformed by CSF-A*β* and CSF-*τ*, in pg/mL.

#### MRI-based Biomarkers

MRI-based biomarkers used in this study correspond to measured of neuro-degeneration available in ADNI at 5 years follow-up. These biomarkers were obtained with the FreeSurfer image analysis suite (version 5.1.0) developed by *Martinos Center for Biomedical Imaging* and freely available at the “*Freesurfer wiki site*” (http://surfer.nmr.mgh.harvard.edu/fswiki). FreeSurfer has been used in several studies dedicated to automatically obtaining MRI-based measures from whole brain or specific regions to sucessfully predict the early conversion from MCI to AD subjects [19, 23, 24, 31, 34, 38, 39]. Technical details of the FreeSurfer processing pipeline have been described in prior publications [40, 41]. Biomarkers with missing values for most samples were discarded. Likewise, we only included the ones correctly processed and available for at least two time points. In this sense, we included an unbalanced longitudinal data of 166 longitudinal MRI-based biomarkers measured at multiple time points: baseline, 6, 12, 18, 24, 36, 48 and 60 months. These biomarkers correspond to cortical volume (CV), cortical thickness average (TA) and the volume estimates of a wide range of sub-cortical structures (SV).

#### Subjects and inclusion criteria

Participants were selected from original ADNI study if they met the following criteria (at the time of the study, April, 2015): (1) Had all selected longitudinal MRI images correctly processed (2) Had completed demographic and neuropsychological data and were clinically diagnosed at each visitation. In total, longitudinal data of 747 subjects (215 CN, 366 LMCI and 166 AD) were studied. Demographic details of the studied cohort are given in Table in S1 Table. Summaries are grouped by gender and correspond to baseline stage. It should be noted that just 164 women and 236 men have available measures of CSF-based biomarkers.

## Methods

**Fig 1:**
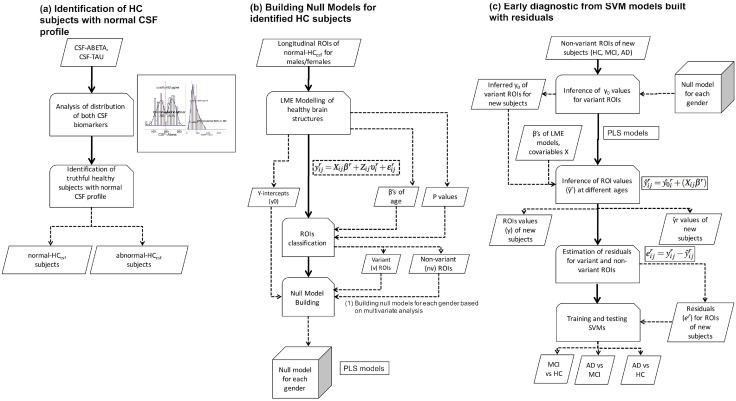
Proposed framework. (A) HC subjects with normal CSF profile are identified from cutoff values calculated from CSF biomarkers distributions. (B) Longitudinal ROIs of these subjects are modelled using LME approach, variant (*vr*) and quasi-variant (*qvr*) ROIs and Y-intercepts (*y*_0_) ROIs values are identified and then null models for both genders are built from these values by applying multivariate modelling. (C) *qvr* ROIs values of new HC, MCI and AD subjects are used within null models to infer the *y*_0_ values of *vr* ROIs. Estimated ROIs values ($\hat{y}$) at different ages are estimated by linear regression by using *y*_0_ and *β* coefficients of age and education. Residuals are calculated as the difference $y-\hat{y}$; and finally, SVM classifiers are trained for subject classification and addressing the early diagnosis problem: HC vs MCI, MCI vs. AD and HC vs AD. The full workflow of last two stages is applied separately for each gender.

This study was divided into three main stages: (1) Subject classification, (2) Building the ageing-based null models, and (3) Residuals-based early prediction of conversion to MCI/AD and HC/MCI/AD classification with SVM classifiers. Fig 1 illustrates a schematic diagram of the proposed framework.

### Classification of Subjects

Since the diagnosis of MCI and AD is progressive, in addition to ADNI clinical assessment established at every visitation (*dx*_*age*_), we aim to control the last ADNI clinical diagnosis of each subject at time of this study, by building a time-invariant diagnosis variable (*dx*_*last*_). This variable labels subjects according the following classes: (1) stable HC (sHC), (2) stable MCI (sMCI), (3) converted to MCI (cMCI), (4)stable AD (sAD) and (5) converted to AD (cAD). Because previous studies have found ADNI participants with abnormal concentrations of CSF-based biomarkers even in healthy elderly subjects [18, 42], we decided to study the subjects with available CSF data (see Table in S1 Table) and to discriminate the ones with normal CSF-profile from the ones with abnormal concentrations. For this propose, we investigated the cut-off values previously established by [18] (CSF-A*β*: 192*pg*/*ml*, CSF-*τ*: 93*pg*/*ml*) [18] from their study with ADNI-independent autopsy-based samples, on our samples at long-term follow-up times (84 months). As many of these subjects have more than one measurement available for both CSF-based biomarkers, the normal or abnormal profile was evaluated for all observations. In this way, we expected to classify these subjects through the other time-invariant feature (*dx*_*csf*_), which combines their *dx*_*last*_ state and their CSF profile. Table 1 gives a brief description of all diagnosis variables used in this study. Once the subjects were characterized according to *dx*_*last*_ and *dx*_*csf*_, two cohorts were established: (1) The null model cohort, integrated by the stable HC subjects with normal CSF profile (normal-HC_csf_); and (2) The early prediction cohort, integrated by the remaining subjects who did not meet the previous condition.

**Table 1:** Classification results with current diagnostic.

Source	Diagnostic variable	Class	Class description
ADNI	*dx*_*bl*_	CN	Control normal at baseline
		LMCI	Late MCI at baseline
		AD	Early probable AD at baseline
	*dx*_*age*_	NL	Subjects diagnosed as stable normal at current visitation
		NL to MCI	Subjects diagnosed as MCI at current visit who previously were NL
		NL to Dementia	Subjects diagnosed as dementia due to AD at current visit who previously were NL
		MCI	Subjects diagnosed as stable MCI at current visit who previously were also MCI
		MCI to Dementia	Subjects diagnosed as dementia due to AD at current visit who previously were MCI
		Dementia	Subjects diagnosed as stable dementia due to AD at current visit who previously were also MCI
Our study	*dx*_*last*_	sHC	Subjects labelled as HC who remained like HC in all follow-up visits (who did not become MCI or AD)
		sMCI	the MCI subject who did not become AD
		cMCI	Subjects initially labelled as HC who subsequently have converted to MCI
		sAD	Subjects who remained like probable or possible AD all the follow-up visits
		cAD	Subjects labelled as HC or MCI who subsequently have converted to probable or possible AD
	*dx*_*csf*_	normal-HC_csf_	sHC subjects with normal CSF profile
		abnormal-HC_csf_	sHC subjects with abnormal CSF profile
		normal-MCI_csf_	sMCI and cMCI subjects with normal CSF profile
		abnormal-MCI_csf_	sMCI and cMCI subjects with abnormal CSF profile
		normal-AD_csf_	sAD and cAD subjects with normal CSF profile
		abnormal-AD_csf_	sAD and cAD subjects with abnormal CSF profile

### Building the ageing-based null models

In this stage, we analyzed data from the null model cohort. Every MRI-based biomarker of the normal-HC_csf_ group was standardized to have zero mean and unit variance. Here, we also refer to an MRI-based biomarker as a Region of Interest (ROI) when we refer to the modelling process.

#### Modelling temporal change in ROIs

To visualize the between-subject and within-group variabilities on normal-HC_csf_, ROIs were represented by quartiles. The change in cortical and subcortical brain regions over 5 years was calculated by applying the LME approach for every ROI. The subject age (*age*) at each observation and the years of education (*educ*) were included as covariates in all models. Because we hypothesize that there are important individual-level effects and believe that subjects have similar rates of change over time, we fitted random intercepts LME models. In this type of model, the measured value of ROI *r* defined as *y*_*ij*_ is assumed to have a set of parameters *β*, fixed across subjects. In addition, for each individual *i*, a set of random parameters *υ*_*i*_ is assigned that models the deviation from the fixed effect *β*. For *i* = 1, …, *n*, each model reads as follows:

$$\begin{array}{c} y_{ij}^{r}=X_{ij}\beta^{r}+Z_{ij}\upsilon_{i}^{r}+\epsilon_{ij}^{r} \end{array}$$ (1)

where, $y_{ij}^{r}$ is the standardized value of ROI *r* measured for the *i*^*th*^ subject in the *j*^*th*^ observation; *i* = 1, …, *n* subjects, *j* = 1, …, *n*_*i*_ available observations for subject *i* and *r* = 1, …, *n*_*r*_, *n*_*r*_ = 166 ROIs. *X*_*ij*_ is a *n*_*i*_ x *p* design matrix, where *p* is the number of covariates (*age*, *educ* and the constant term of 1’s) on the *j*^*th*^ observation of *i*^*th*^ subject. *β*^*r*^ is the *p* x 1 vector of unknown fixed effects or regressor’s coefficients, which are: $\beta_{1}^{r}$ (coefficient for constant term or *Intercept*), $\beta_{a}^{r}$ (coefficient for *age*) and $\beta_{e}^{r}$ (coefficient for *educ*). *Z*_*ij*_ is a known design matrix of size *n*_*i*_ x *q*, where *q* is the number of random effects for the *j*^*th*^ observation of subject *i*. $\upsilon_{i}^{r}$ is the *q* × 1 vector of unknown random effects coefficients ∼*N*_*q*_(0, *ψ*) for subject *i* measured for ROI *r*. $\epsilon_{ij}^{r}$ is the *n*_*i*_ *x*1 residual vector of errors ∼*N*_*ni*_(0, *σ*^2^ *λ*_*i*_) for the *j*^*th*^ observation in subject *i* measured for ROI *k*. *ψ* is the *q* × *q* covariance matrix for the random effects. 0, *σ*^2^ *λ*_*i*_ is the *n*_*i*_ x *n*_*i*_ covariance matrix for the errors in subject *i*.

LME modelling was performed using the *lme*4 package available for R [43]. See S1 Appendix for further detail of the specific LME approach.

#### Identification of variant and quasi-variant ROIs

The predictor estimates of LME models were interpreted the same way as the coefficients from a traditional regression. For instance, a one year increase in the regressor *age* corresponded to the effect of *age* (*βa*) increase or decrease in the outcome. Taking into account the *β*_*a*_’s, we classified as variant (*vr*) ROIs the ones that had both an annual change greater than 1% on ROI standard deviation and a significant change at *p*-values ≤ 0.05. The ROIs with annual change less than or equal to 1% were considered as quasi-variant (*qvr*), where all of these did not have a significant change.

#### Inference of variant ROIs y-intercept values from quasi-variant ROIs

The Y-intercept value (*y*_0_) from each ROI, which represents the subject-specific ROIs measure at basal stage (*age* = 0), was directly obtained from all LME models. We assumed that for healthy elderly people, the *qvr* ROIs values remains basically the same along time, even at basal stage, which is not true in the *vr*. We also assumed there are a strong correlation between the *y*_0_ set of both ROI types. Therefore, we built the HC null model based on the Partial Least Squares Regression (PLSR) algorithm [44] to infer the set of *y*_0_’s for *vr* ROIs in function of the set of *y*_0_’s for *qvr* ROIs. PLSR is a linear algorithm particularly useful to analyze data with strongly collinear (correlated), noisy, and numerous predictors variables, and also simultaneously model several response variables [45]. There are several algorithms proposed to implement the PLSR model. In this study, we use the *kernel* algorithm [46] available in the *pls* package available for R [47]. To determine the optimal number of components to take into account, it was used leave-one-out (LOO) cross-validation method available in this package. LOO calculates potential models excluding one observation at a time. See S1 Appendix for a short PLSR description.

### Early disease prediction based on residuals

In this stage, we addressed the subject classification and early disease prediction problems using data from the early prediction cohort (HC, MCI and AD), unused in the previous stage. ROIs of these subjects were standardized according to the mean and standard deviation of the null model cohort.

#### Inference of age-associated ROIs values for new subjects

For each subject, the *y*_0_’s of *vr* ROIs were inferred from the *y*_0_’s of *qvr* ROIs using the same PLSR model described in previous section. Once the *y*_0_ set for both ROIs types was completed, we used these values in combination with the vector of coefficients *β*, see Eq (1) and the observed covariates (*age* and *educ*), to infer the expected values of each ROI (${\hat{y}}_{ij}$) according to Eq (2). Here,the inferred value represents the value that should measure the ROI at *age* ≠ 0 whether the subject is healthy or not.

$$\begin{array}{c} {\hat{y}}_{ij}^{r}={\hat{y_{0}}}_{i}^{r}+\left(X_{ij}\beta^{r}\right) \end{array}$$ (2)

where ${\hat{y}}_{ij}^{r}$ represents the inferred or predicted value of the *r*^*th*^ ROI variant on the *j*^*th*^ observation for the *i*^*th*^ subject. *X*_*ij*_ is the design matrix with observed covariates at *age* ≠ 0 without the constant term. *β*^*r*^ is the vector of calculated fixed effects of ROI *r* obtained from its LME model, according Eq (1).

#### Computing the residuals of variant ROIs

In order to get a meaningful deviation value of ROIs from an inferred healthy subject-specific trend, the difference between the estimated ($\hat{y}$) and the true ($\hat{y}$) ROIs values, here called residual (*e*), was computed. The residuals $e_{ij}^{r}$ for each ROI were calculated as following:

$$\begin{array}{c} e_{ij}^{r}=y_{ij}^{r}-{\hat{y}}_{ij}^{r} \end{array}$$ (3)

where $y_{ij}^{}$ and ${\hat{y}}_{ij}$ are the observed and inferred values, respectively, for each ROI *r* measured on the *i*^*th*^ subject in the *j*^*th*^ observation. The *e*’s for all ROIs were stored in a matrix **E**.

In S1 Appendix we describe a hypothetical example of how we have used the LME and PLSR approaches to infer the ROI values at basal stage and over time; and finally, the residuals.

#### Diagnosis prediction using Support Vector Machines

In this stage, we used the matrix of residuals **E** to address two problems. The first one was focused on the subject classification, where the vector of class labels, used as the outcome, was the diagnostics at the time of the visit, *dx*_*age*_ (Table 1). The second problem was the early disease prediction, here, we trained a classifier that predicts the future diagnosis of the subjects given their current clinical tests, i.e. what is the expected diagnostic of the subject some years after the current visit. The vector of class labels used to train this classifier was *dx*_*last*_ (Table 1). In this case, as the feature set used was obtained in previous visits of the subject, the classifier learns to predict the future outcome of a subject, given his present state.

For both problems, we performed three experiments focused on the binary classification problems: (1) HC vs AD, (2) HC vs MCI and (3) MCI vs AD. The MCI vs AD experiment in the early prediction problem was focused on addressing the problem of prodromal stage of AD, by comparing the sMCI (stable MCI over all visitations) with the subject initially diagnosed as MCI who became to AD over time (cAD). Likewise, for each problem, two configurations of the feature set have been tested. Let *E*_*v*_ be the subset of matrix *E* where only the columns of either the *vr* ROIs or the *qvr* ROIs whose residuals are different from zero were included. The first feature set used in each experiment, *F*_1_, included the residuals from matrix *E*_*v*_ plus the *age* (age of the subject at the time of the visit). The second feature set used, *F*_2_, includes the information in *F*_1_ together with the results from the CDR global score (CDRGLOBAL) and MMSE tests. The goal of testing two training sets was to assess the influence of the neuropsychological tests in the quality of diagnostic as opposed to the use of ROI residuals with age only.

The classification method used to carry out each experiment is a Support Vector Machine (SVM), configured with a Gaussian radial kernel and a misclassification cost parameter *C* = 1. Each experiment has been evaluated using a ten-fold cross validation with a specific constraint: as ADNI is a longitudinal database, there may be multiple samples per subject (multiple visits). The subjects were assigned to either the training or the test subsets, in order to perform a fair evaluation. On each cross validation run, 60% of the subjects weren assigned to the training subset and the remaining 40% to the test subset.

Because, it is known that the rate of atrophy increases with age, for studying the development of our method for early disease prediction, SVM classifiers were built on age groups. The ADNI data used to evaluate the classification for males and females are summarized in tables in S2 and S3 Tables, respectively. These tables provide demographic information and the number of measurements and diagnoses by each age group.

Since previous studies have suggested that there are gender differences in brain atrophy with aging [8, 9, 48–50], the full workflow in building null models and early diagnostic from SVM models was applied separately for each gender.

## Results

### Healthy elderly subjects with normal CSF profile

**Fig 2:**
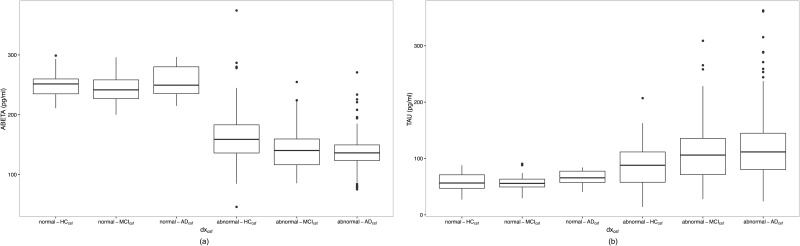
Boxplot of CSF biomarkers concentrations for *dx*_*csf*_ diagnostic groups. (a) CSF-*Aβ*1 − 42; and (b) CSF-*τ*.

Analysis of CSF-A*β* and CSF-*τ* biomarkers showed us that their distributions were not normal for all diagnosis groups (see figure in S2 Fig). A bimodal distribution was observed in CSF-A*β* levels within each diagnosis group. CSF-*τ* values in CN group showed a unimodal normal distribution. We confirmed cut-off values determined by [18], classifying as normal the CSF profile of subjects who fulfilled both conditions: CSF-A*β* > = 192*pg*/*ml* and CSF-*τ* ≤ 93*pg*/*ml*. From CSF profiles, we identified 46 normal-HC_csf_, 33 normal-MCI_csf_, 11 normal-AD_csf_, 63 abnormal-HC_csf_, 75 abnormal-MCI_csf_ and 172 non-*AD*_*csf*_ subjects. Figure in S3 Fig shows CSF-*τ* concentration versus CSF-A*β* concentration for these six groups, where the blue dots represent the HC subjects with normal CSF profile used to build the null models for men and women. Furthermore, in Fig 2 is shown the last available measurements of CSF biomarkers concentrations for the studied ADNI subjects grouped according *dx*_*csf*_. An increase in *τ* values was observed when comparing normal-HC_csf_ and subjects with abnormal CSF-profile, and when comparing abnormal-HC_csf_ with MCI and AD subjects with abnormal profile. With respect to A*β* levels, we observed a reduction of levels when comparing normal-HC_csf_ with abnormal-HC_csf_ and the remaining groups with abnormal profile.

In total, 226 samples were available for the 46 normal-HC_csf_ subjects (males: 22, females: 24). Table 2 provides their demographic information at baseline. Age, education and MMSE scores were similar across gender, but CSF concentrations and CDRGLOBAL were different between men and women. Furthermore, only three subjects (2 males and 1 female) were carriers of APOE-*ϵ*4 at allele 1. More detail of demographic and cognitive measures are given in S1 Appendix.

**Table 2:** Baseline statistical descriptors of HC subjects selected for null models building.

	Female	Male
	*N* = 24	*N* = 22
**age**	71.2 75.1 77.7 (74.7± 5.3)	70.4 71.8 74.0 (72.7± 6.0)
**education**	65.0 70.0 80.0 (0.73± 0.09)	70.0 82.5 90.0 (0.81±0.15)
**APOE-*ϵ*4**		
0	95.8% $\frac{23}{24}$	90.9% $\frac{20}{22}$
1	4.2% $\frac{\;1}{24}$	9.1% $\frac{\;2}{22}$
**MMSE**	29.0 29.0 30.0 (29.25± 0.67)	28.0 29.0 30.0 (28.54± 1.50)
**CDRGLOBAL**		
0	100% $\frac{24}{24}$	82% $\frac{18}{22}$
0.5	0% $\frac{\;0}{24}$	18% $\frac{\;4}{22}$
**CSF-A*β***	234.9 248.5 256.0 (247.23± 20.14)	235.0 257.9 268.2 (253.22± 21.69)
**CSF-*τ***	47.9 55.260.9 (55.41± 15.10)	47.0 59.9 73.8 (59.84± 16.83)

Values of continuous variables are represented by the lower, the median and the upper quartiles; and the *mean*± standard deviation in parentheses.

### Ageing-based variant (*vr*) and quasi-variant (*qvr*) ROIs

A study of within-subject and between-subject variabilities, using the box plots representation, showed us that there are significant between-subject variability and strong gender effect for several ROIs; for example, the left hippocampal volume (see Figure in S4 Fig). However, for other ROIs their change was not very evident. From LME modelling, we identified 97 *vr* ROIs for males and 109 *vr* ROIs for females. Regarding quasi-variant ROIs, we found 69 *qvr* for males and 57 *qvr* for females. The figure in S5 Fig shows examples of fitted LME models for both types of ROIs within each gender. In a similar way to these examples, the *β*_*a*_’s of the remaining *vr* ROIs were observed as being close to zero, but for *qvr* ROIs, their slopes were not. The figure in S6 Fig shows the summary of ageing-associated differences of biomarkers in men and women. The results suggest important ageing-related reductions in neocortical and subcortical regions and ventricular expansion, with several gender-specific significances. Reductions are observed in the most of cortical volumes and cortical thickness, where men and women showed a similar degree of global thinning. Some of these regions showed prominent atrophy while others showed a more conservative change; for example, the entorhinal volume was observed to be significantly reduced in females but not in males. In relation to the subcortical regions, we observed several gender-specific differences, some of these are: significant volumetric reductions in both hemispheres of hippocampal volume for both males and females; ventricular expansion in both genders, but just the change of fourth ventricle volume was not significant; the volume of the optic chiasm region was significantly increased in females, but it was not significant in males; a reduction in amygdala volume was significant in both hemispheres of the male brain but it was not significant in females; and, in general, the volume of bilateral corpus callosum regions are observed as thinned, but without significant differences between the genders.

### Subject classification based on ROI residuals

The table in S4 Fig shows the average prediction accuracy (ACC), sensitivity (SEN) and specificity (SPE) of the test for both the *F*_1_ and *F*_2_ training set configurations. Although the performance of models with the *F*_2_ set was the best for all cases, the experiments performed on the *F*_1_ set had also good results. Table 3 compares the prediction accuracy of the proposed method (by averaging the accuracy for both genders) with existing methods, which have been evaluated on the ADNI dataset and other data sources. Note that we only showed the most significant results of those studies, mainly focusing on MRI and its combination with other features.

**Table 3:** Comparison of methods performances focused on subject classification (%).

Method	AD vs HC			MCI vs HC			cAD vs sMCI			Data used
	ACC	SEN	SPE	ACC	SEN	SPE	ACC	SEN	SPE	
Kloeppel et al. [30]	95.0	95.0	95.0	-	-	-	-	-	-	MRI
Vemuri et al. [3]	-	86.0	86.0	-	-	-	-	-	-	MRI
	-	88.0	90.0	-	-	-	-	-	-	MRI,age,gender
	-	86.0	92.0	-	-	-	-	-	-	MRI,age,gender,APOE
Cui et al. [19]	-	-	-	-	-	-	67.13	96.43	48.28	NM,CSF,MRI
	-	-	-	-	-	-	62.24	92.86	42.53	NM,MRI
	-	-	-	-	-	-	62.24	57.14	65.52	MRI
Cuingnet et al. [31]	-	81.0	95.0	-	-	-	-	57.0	78.0	MRI
Zhang et al. [25]*	93.3	-	-	83.2	-	-	73.9	68.6	73.6	MRI,PET,CSF
Suk et al. [28]	95.9	-	-	85.0	-	-	75.8	-	-	MRI,PET
Jie et al. [26]	95.03	94.90	95.00	79.27	85.86	66.64	68.94	64.65	71.79	MRI,FDG-PET
Gaser et al. [22]	-	-	-	-	-	-	75.00	71.00	84.00	MRI-based age
Spulber et al. [23]	88.4	86.1	90.4	-	-	-	67.7	69.6	66.8	MRI-based index
Aguilar et al. [24]	-	92.0	75.0	-	-	-	-	92.0	47.0	MRI-based index
Liu et al. [27]	94.37	94.71	94.04	78.8	84.85	67.06	67.83	64.88	70.0	MRI,PET
Suk et al. [29]	92.38	91.54	94.56	84.24	99.58	53.79	72.42	36.70	90.98	MRI
	93.35	94.65	95.22	85.67	95.37	65.87	75.92	48.04	95.23	MRI,PET
*Proposed method (*F*_1_)	**89.22**	**90.73**	**86.85**	**77.08**	**95.53**	**26.98**	**70.30**	**64.26**	**75.14**	MRI-based residuals, age
*Proposed method (*F*_2_)	**94.11**	**96.54**	**90.28**	**83.77**	**93.55**	**57.55**	**76.72**	**70.77**	**81.62**	MRI-based residuals, age, MMSE,CDRGLOBAL

* Results of this method correspond to the average of performance recorded for men and women. MRI, Magnetic Resonance Imaging-based features; CSF; Cerebral Spinal Fluid-based biomarkers; NM: Neuro-psychological measures; PET, Positron Emission Tomography-based features; FDG-PET, [18F]fluorodeoxyglucose uptake measured in PET; MRI-based age, individual estimated age computed from MRI images; MRI-based index, individual severity index computed from MRI images; MMSE, Mini-Mental Clinical Dementia Rating; CDRGLOBAL, CDR Global Score.

### Early prediction of subject diagnostics based on ROI residuals

The table in S5 Table shows the performance of models built for early prediction of disease progression. As in previous results, although models built with the *F*_2_ configuration have obtained slightly better accuracies than the ones built on the *F*_1_ configuration, both performances are very satisfactory. Table 4 shows the average number of years this method leads in predicting the subject diagnosis. This advancement in the prediction was only possible in subjects whose *dx*_*age*_ (diagnostic at each visitation) was different from their *dx*_*last*_ diagnosis (last known diagnostic). Therefore, only these subjects were taken into account and some age groups lack enough data to be shown in this table. For females, the prediction of conversion from MCI to AD was up to 1.9 years earlier (60–64 age group); and for males, up to 1.73 years earlier (80–84 age group). However, the greatest lead was obtained in the early prediction from HC to MCI in females, this being 2.89 years earlier.

**Table 4:** Last known diagnostic prediction advancement.

		Advancement for early prediction in years					
	Experiment	60–64 yrs.	65–69 yrs.	70–74 yrs.	75–79 yrs.	80–84 yrs.	85–89 yrs.
Females	AD vs HC	1.772 (85.73)	1.596(86.05)	1.335(86.79)	1.745(88.93)	1.523(90.21)	1.245(89.67)
	MCI vs HC	N/A	N/A	2.888(74.42)	2.071(76.08)	2.381(78.40)	N/A
	AD vs MCI	1.982(69.23)	1.539(70.04)	1.307(69.55)	1.751(71.37)	1.509(72.41)	1.433(71.28)
Males	AD vs HC	1.232(84.27)	1.801(85.30)	1.667(86.91)	1.327(87.75)	1.775(89.44)	1.006(89.52)
	MCI vs HC	1.257(72.47)	N/A	1.739(77.29)	2.529(78.32)	2.686(78.59)	1.439(79.81)
	AD vs MCI	1.448(68.95)	1.697(69.44)	1.679(70.22)	1.372(71.18)	1.733(73.46)	1.011(73.06)

N/A means there are not enough samples in the age interval for binary classification. (ACC) represents the prediction accuracy of *F*_2_ method in %

Along with the time advancement in the prediction, Table 4 also shows the average prediction accuracy for that differential diagnostic stratified by gender and age group. Note that differences between accuracies of Tables 3 and 4 are due to the last one included a few samples, namely those that fit the conditions of five-year age group stratification and whose last known diagnostic differs from the diagnostic at the time of the test in the given age group.

## Discussion and Conclusions

This study has presented a framework for building ageing-associated null models from longitudinal MRI-based biomarkers, as well as subject classification and early diagnosis from residuals calculated through these null models.

Analysis of CSF biomarkers distributions allowed us to confirm the cut-offs values established by [18] and to establish criteria for CSF profiling. We found subjects clinically diagnosed as CN at baseline with abnormal CSF profile, which is consistent with previous studies where the presence of possible AD pathology has been found in ADNI control subjects [18, 51, 52]. These findings may be because ADNI subject diagnosis is made independently of the CSF biomarkers values (see S1 Appendix).

We modelled the longitudinal change of an extensive set of MRI-based biomarkers obtained from cortical and subcortical regions. Studies have shown that early diagnosis methods using the whole brain or the whole cortex reached higher specificity (over 90%) than those based on the specific regions like the hippocampus (from 63% to 84%) [31, 53, 54]. LME modelling allowed to classify biomarkers as variant or quasi-variant ROIs, and to build null models for ageing-related changes in men and women. As in previous studies [6, 8, 9], our results (see Figure in S6 Fig) show that part of these changes occur in brain areas related with AD. We found reductions for most thickness and volume biomarkers of cortical regions [8] in both genders. Similar changes were also found in subcortical regions including the greater atrophy in the hippocampus and regions of the corpus callosum.

Null models were carried out by making assumptions of correlation between the y-intercepts values (at basal stage) of variant and quasi-variant ROIs, where the first ones were explained in function of last ones using PLSR. By using these null models and LME *β* coefficients, we calculated residuals, which were established as differences between the observed ROIs and age-related inferred ROIs. These residuals were computed for new cohort of HC, MCI and subjects; and used for training and testing SVM models to address subject classification and early disease prediction.

As of the date of this study, we were unable to find studies where ageing-related null models and residuals-based classifiers were applied to early diagnosis. The performance obtained in all experiments suggests that the proposed method of obtaining the ROI residuals and their use to train SVM predictors is useful to support the early diagnosis problem, the fundamental challenge in AD research. LME modelling of MRI-based biomarkers was only applied by fitting age and years of education, but future work could assess the impact of different available feature types such as other functional neuroimaging biomarkers, genetic factors, biological markers and other clinical and neuropsychological assessment.

The main contributions of the residual-based classifier presented in this paper are: (a) From a longitudinal study of 5 years follow-up, the ability to predict the future diagnostic of the subjects up to 2.88 years earlier than the standard clinical procedure. (b) Use of relatively common clinical tests such as MRI and neuropsychological tests, as opposed to methods that rely on more expensive or invasive tests such as PET-based, CSF-based and Genotype-based biomarkers. (c) HC vs AD: highest sensitivity among the state of the art methods; a classification accuracy of 94%, higher than all MRI-only methods except for [30]. (d) sMCI vs cAD: highest classification accuracy among the state of the art methods. (e) In most experiments, the sensitivity (the ability of a predictor to correctly classify a subject as ‘diseased’) was higher than the specificity (the ability of predictor to correctly classify a subject as ‘disease-free’). This may be due to the fact that ADNI clinical diagnosis is based on neuro-psychological tests, but neurodegeneration occurs many years before the onset of clinical symptoms. Possibly, residual-based SVM predictors may determine that subjects are into early stages of disease (MCI and prodromal AD) but this finding is not consistent with clinical diagnosis because the subject does not yet present clinical symptoms. Abnormalities of CSF profile observed on several subjects diagnosed as HC and MCI (see Figure in S3 Fig) may support this hypothesis.

The use of MMSE and CDRGLOBAL tests (*F*_2_ method) yields significantly better results than using the residuals alone (*F*_1_ method). These are two of the most common neuropsychological tests routinely applied to patients in the primary clinical practice. However this should not be seen as a mere contribution of MMSE and CDRGLOBAL, as these tests on their own have several limitations such as relatively low specificity and reliability. However, they complement and enhance the present method here without adding a significant cost or invasive clinical tests.

Despite promising results, there are several limitations to our study. Firstly, data used here correspond to research participants, who meet the inclusion and exclusion criteria established by ADNI, and thus are not from general population. Secondly, the available observations of CSF biomarkers do not correspond in number or time points with the available MRI-based observations. In most cases we had just CSF values at the baseline stage, so it is impossible to track the reliability of CSF profile at the final stages. Due to this, it is important to emphasize that subject classifications based on CSF profile are applicable to the ADNI study subjects, but not necessarily to individuals in other settings. Thirdly, like CSF biomarkers, we had substantial missing data for MRI-based biomarkers measured for 46 HC subjects during the 60-month follow-up period. Although a LME approach can handle missing time points within individuals, this issue may limit our ability to make inferences about the age-related changes in these biomarkers. Finally, the proposed methodology to obtain a null model of healthy elderly people is still at the early stages of the development and evaluation process, it is necessary to test the null model in populations different from those used in model development.

## Supporting Information

S1 Appendix Brief Data and Methods Description. (PDF)

S1 FigExample of LME modelling for hypothetical variant (*vr*) and quasi-variant(*qvr*) ROIs.HC and AD are hypothetical subjects. *P*_1_, *P*_2_ and *P*_3_ are observations of each ROI *y* at three different ages (*a*_1_, *a*_2_ and *a*_3_). Black lines, healthy population regression line calculated from LME. ${\hat{y}}_{0}$, vertical y-intercept value of healthy population. Blue and red lines, individual regression lines estimated by assuming both as healthful. Points ${\hat{P}}_{1}$, ${\hat{P}}_{2}$ and ${\hat{P}}_{3}$, inferred $\hat{y}$’s for the three ages.${\hat{y}}_{HC_{0}}$ and ${\hat{y}}_{AD_{0}}$, the subject-specific y-intercepts estimated for HC and AD subjects, respectively. ${\hat{y}}_{HC_{0}}$ and ${\hat{y}}_{AD_{0}}$ of *vr* ROI are inferred from the ${\hat{y}}_{HC_{0}}$ and ${\hat{y}}_{AD_{0}}$ of *qvr* ROI through PLSR model. *β*_*a*_, slope or rate change of standard deviation of ROI per unit of age. *ϵ*_*HC*1_, *ϵ*_*HC*2_, *ϵ*_*HC*3_, *ϵ*_*AD*1_, *ϵ*_*AD*2_ and *ϵ*_*AD*3_, the residuals of each observation with respect to the estimated individual regression lines.(PDF)

S2 Fig Distribution of CSF-based biomarkers at follow-up 84 months. (a) CSF-A*β*. (b) CSF-*τ*. △: 8 of 113 CN subjects were converted to MCI and 1 CN subject was converted to AD. ○: 88 of 94 LMCI were converted to AD and 5 were re-converted to HC at follow-up visits. Dotted vertical lines within each diagnosis are the determined cutoff concentrations. Figure shows us that several subjects with abnormal CSF profile classified as CN by ADNI at baseline clinical assessment were converted to MCI or AD (triangle dots) along time. Likewise, some subjects classified like ‘LMCI’ by ADNI were converted to AD later (open circles).
(PDF)

S3 Fig CSF-A*β* vs. CSF-*τ* concentration available at last subject’s observations. Dots represent the last CSF biomarker measured for subjects available at April, 2015. Vertical and horizontal dashed lines split normal CSF-profile from abnormal profile. Null models for characterization of healthy brain structures were built from samples labelled with blue dots.
(PDF)

S4 Fig Boxplot of trajectory of left hippocampal volume for normal-HC_csf_ subjects. High between-subject variability is evident, e.g., by comparing subject 099_*S*_0533 with subject 133_*S*_0488. Likewise, there is a strong indication of gender effect over hippocampal volume, female volumes are less than the male ones. Note that we standarized every MRI-based biomarker to have zero mean and unit variance.
(PDF)

S5 Fig Examples of variant and quasi-variant ROIs per normal-HC_csf_ subjects stratified by gender. (a) Left hippocampal volume classified as variant (*vr*) ROI; where the slope of trajectories is not close to zero. (b) Left caudate volume classified as quasi-variant (*qvr*) ROI; where the slope of trajectories is close to zero. Note that for both regions, the y-intercept values vary between subjects, but the slope value of each ROI is the same for all subjects.
(PDF)

S6 Fig Characterization of ageing-based variant (*vr*) and quasi-variant (*qvr*) ROIs. (a) Males. (b) Females. Biomarkers are coloured according their change type: blue for *vr* regions and red for the *qvr* ones. The size of biomarkers with significant *P*-value ≤ 0.05 are bigger than the not significant. Dotted vertical lines separate the increased biomarkers (positive beta values) from the reduced ones (negative beta values). ROIs are represented according to their location into brain hemisphere (lh:left, rh: right or bilateral). SV: Subcortical Volume, CV: Cortical volume, SA: Surface Area. lh: left hemisphere. rh: right hemisphere.
(PDF)

S1 Table Statistical descriptors of studied ADNI cohort at baseline. (PDF)

S2 Table Statistical descriptors of males used to build the SVM. (PDF)

S3 Table Statistical descriptors of females used to build the SVM. (PDF)

S4 Table Performances of classification for current diagnostic. (PDF)

S5 Table Performances of classification for last known diagnostic. (PDF)

## References

[1] Jack CR, Knopman DS, Jagust WJ, Shaw LM, Aisen PS, Weiner MW, et al Hypothetical model of dynamic biomarkers of the Alzheimer’s pathological cascade. Lancet Neurol. 2010 1;9(1):119–128. Available from: 10.1016/S1474-4422(09)70299-6. 20083042 PMC2819840

[2] Dubois B, Hampel H, Feldman HH, Scheltens P, Aisen P, Andrieu S, et al Preclinical Alzheimer’s disease: Definition, natural history, and diagnostic criteria; 2016 10.1016/j.jalz.2016.02.002 27012484 PMC6417794

[3] Vemuri P, Gunter JL, Senjem ML, Whitwell JL, Kantarci K, Knopman DS, et al Alzheimer’s disease diagnosis in individual subjects using structural MR images: validation studies. Neuroimage. 2008 2;39(3):1186–1197. Available from: 10.1016/j.neuroimage.2007.09.073. 18054253 PMC2390889

[4] Frisoni GB, Fox NC, Jack CR, Scheltens P, Thompson PM. The clinical use of structural MRI in Alzheimer disease. Nature reviews Neurology. 2010;6(2):67–77. Available from: http://www.nature.com.ezproxy.usc.edu.au:2048/nrneurol/journal/v6/n2/full/nrneurol.2009.215.html. 10.1038/nrneurol.2009.215 20139996 PMC2938772

[5] Saykin AJ, Shen L, Foroud TM, Potkin SG, Swaminathan S, Kim S, et al Alzheimer’s Disease Neuroimaging Initiative biomarkers as quantitative phenotypes: Genetics core aims, progress, and plans. Alzheimer’s & dementia: the journal of the Alzheimer’s Association. 2010 5;6(3):265–73. Available from: http://www.pubmedcentral.nih.gov/articlerender.fcgi?artid=2868595{&}tool=pmcentrez{&}rendertype=abstract. 10.1016/j.jalz.2010.03.013 20451875 PMC2868595

[6] Fjell AM, Walhovd KB, Fennema-Notestine C, McEvoy LK, Hagler DJ, Holland D, et al One-year brain atrophy evident in healthy aging. J Neurosci. 2009 12;29(48):15223–15231. Available from: 10.1523/JNEUROSCI.3252-09.2009. 19955375 PMC2827793

[7] Peters R. Ageing and the brain. Postgraduate medical journal. 2006 2;82(964):84–8. Available from: http://www.pubmedcentral.nih.gov/articlerender.fcgi?artid=2596698{&}tool=pmcentrez{&}rendertype=abstract. 10.1136/pgmj.2005.036665 16461469 PMC2596698

[8] Salat D, Buckner RL, Snyder AZ, Greve DN, Desikan RS, Busa E, et al Thinning of the cerebral cortex in aging. Cerebral Cortex. 2004;14:721–730. 10.1093/cercor/bhh032 15054051

[9] Jiang J, Sachdev P, Lipnicki DM, Zhang H, Liu T, Zhu W, et al A longitudinal study of brain atrophy over two years in community-dwelling older individuals. Neuroimage. 2014 2;86:203–211. Available from: 10.1016/j.neuroimage.2013.08.022. 23959201

[10] Liu Y, Julkunen V, Paajanen T, Westman E, Wahlund LO, Aitken A, et al Education increases reserve against Alzheimer’s disease-evidence from structural MRI analysis. Neuroradiology. 2012;54(9):929–938. 10.1007/s00234-012-1005-0 22246242 PMC3435513

[11] Morris GP, Clark IA, Vissel B. Inconsistencies and controversies surrounding the amyloid hypothesis of Alzheimer’s disease. Acta Neuropathol Commun. 2014;2:135 Available from: 10.1186/s40478-014-0135-5. 25231068 PMC4207354

[12] Wolfe MS. The role of tau in neurodegenerative diseases and its potential as a therapeutic target. Scientifica. 2012;2012:796024 Available from: http://www.pubmedcentral.nih.gov/articlerender.fcgi?artid=3820460{&}tool=pmcentrez{&}rendertype=abstract. 10.6064/2012/796024 24278740 PMC3820460

[13] Morris J. The Clinical Dementia Rating (CDR): current version and scoring rules. Neurology. 1993;43:2412–2414. 10.1212/WNL.43.11.2412-a 8232972

[14] Folstein M, Folstein S, McHugh P. Mini-mental state. A practical method for grading the cognitive state of patients for the clinician. Journal of Psychiatric Research. 1975;12(3):189–198. 10.1016/0022-3956(75)90026-6 1202204

[15] Mueller SG, Weiner MW, Thal LJ, Petersen RC, Jack CR, Jagust W, et al Ways toward an early diagnosis in Alzheimer’s disease: the Alzheimer’s Disease Neuroimaging Initiative (ADNI). Alzheimers Dement. 2005 7;1(1):55–66. Available from: 10.1016/j.jalz.2005.06.003. 17476317 PMC1864941

[16] Mueller SG, Weiner MW, Thal LJ, Petersen RC, Jack C, Jagust W, et al The Alzheimer’s disease neuroimaging initiative. Neuroimaging Clin N Am. 2005 11;15(4):869–77, xi–xii. Available from: 10.1016/j.nic.2005.09.008. 16443497 PMC2376747

[17] Albert MS, DeKosky ST, Dickson D, Dubois B, Feldman HH, Fox NC, et al The diagnosis of mild cognitive impairment due to Alzheimer’s disease: recommendations from the National Institute on Aging-Alzheimer’s Association workgroups on diagnostic guidelines for Alzheimer’s disease. Alzheimers Dement. 2011 5;7(3):270–279. Available from: 10.1016/j.jalz.2011.03.008. 21514249 PMC3312027

[18] Shaw LM, Vanderstichele H, Knapik-Czajka M, Clark CM, Aisen PS, Petersen RC, et al Cerebrospinal fluid biomarker signature in Alzheimer’s disease neuroimaging initiative subjects. Ann Neurol. 2009 4;65(4):403–413. 10.1002/ana.21610 19296504 PMC2696350

[19] Cui Y, Liu B, Luo S, Zhen X, Fan M, Liu T, et al Identification of conversion from mild cognitive impairment to Alzheimer’s disease using multivariate predictors. PLoS One. 2011;6(7):e21896 10.1371/journal.pone.0021896 21814561 PMC3140993

[20] Franke K, Ziegler G, Klöppel S, Gaser C. Estimating the age of healthy subjects from T1-weighted MRI scans using kernel methods: exploring the influence of various parameters. Neuroimage. 2010 4;50(3):883–92. Available from: http://www.sciencedirect.com/science/article/pii/S1053811910000108. 20070949 10.1016/j.neuroimage.2010.01.005

[21] Franke K, Gaser C. Longitudinal changes in individual BrainAGE in healthy aging, mild cognitive impairment, and Alzheimer’s disease. Journal of Gerontopsychology and Geriatric Psychiatry. 2012;25(4):235–45.

[22] Gaser C, Franke K, Klöppel S, Koutsouleris N, Sauer H. BrainAGE in Mild Cognitive Impaired Patients: Predicting the Conversion to Alzheimer’s Disease. PLoS ONE. 2013;8(6). 10.1371/journal.pone.0067346 23826273 PMC3695013

[23] Spulber G, Simmons A, Muehlboeck JS, Mecocci P, Vellas B, Tsolaki M, et al An MRI-based index to measure the severity of Alzheimer’s disease-like structural pattern in subjects with mild cognitive impairment. Journal of internal medicine. 2013 4;273(4):396–409. Available from: http://www.pubmedcentral.nih.gov/articlerender.fcgi?artid=3605230{&}tool=pmcentrez{&}rendertype=abstract. 10.1111/joim.12028 23278858 PMC3605230

[24] Aguilar C, Muehlboeck JS, Mecocci P, Vellas B, Tsolaki M, Kloszewska I, et al Application of a MRI based index to longitudinal atrophy change in Alzheimer disease, mild cognitive impairment and healthy older individuals in the AddNeuroMed cohort. Frontiers in aging neuroscience. 2014 1;6:145 10.3389/fnagi.2014.00145 25071554 PMC4094911

[25] Zhang D, Shen D, Alzheimer’s Disease Neuroimaging Initiative. Multi-Modal Multi-Task Learning for Joint Prediction of Multiple Regression and Classification Variables in Alzheimer’s Disease. Neuroimage. 2012;59(2):895–907. 10.1016/j.neuroimage.2011.09.069 21992749 PMC3230721

[26] Jie B, Zhang D, Cheng B, Shen D. Manifold regularized multi-task feature selection for multi-modality classification in Alzheimer’s disease In: Medical Image Computing and Computer-Assisted Intervention–MICCAI 2013. Springer; 2013 p. 275–283. 10.1007/978-3-642-40811-3_35 PMC4109068 24505676

[27] Liu F, Wee CY, Chen H, Shen D. Inter-modality relationship constrained multi-modality multi-task feature selection for Alzheimer’s Disease and mild cognitive impairment identification. NeuroImage. 2014;84:466–475. 10.1016/j.neuroimage.2013.09.015 24045077 PMC3849328

[28] Suk HI, Shen D. Deep Learning-Based Feature Representation for AD/MCI Classification. Med Image Comput Comput Assist Interv. 2013;16(2):583–590. 24579188 10.1007/978-3-642-40763-5_72 PMC4029347

[29] Suk HI, Lee SW, Shen D, Initiative ADN, et al Hierarchical feature representation and multimodal fusion with deep learning for AD/MCI diagnosis. NeuroImage. 2014;101:569–582. 10.1016/j.neuroimage.2014.06.077 25042445 PMC4165842

[30] Klöppel S, Stonnington CM, Chu C, Draganski B, Scahill RI, Rohrer JD, et al Automatic classification of MR scans in Alzheimer’s disease. Brain. 2008 3;131(Pt 3):681–689. Available from: 10.1093/brain/awm319. 18202106 PMC2579744

[31] Cuingnet R, Gerardin E, Tessieras J, Auzias G, Lehéricy S, Habert MO, et al Automatic classification of patients with Alzheimer’s disease from structural MRI: A comparison of ten methods using the ADNI database. Multivariate Decoding and Brain Reading. 2011;56(2):766–781. Available from: http://www.sciencedirect.com/science/article/pii/S1053811910008578. 10.1016/j.neuroimage.2010.06.013 20542124

[32] Petersen RC, Aisen PS, Beckett LA, Donohue MC, Gamst AC, Harvey DJ, et al Alzheimer’s Disease Neuroimaging Initiative (ADNI). Neurology. 2010;74(3):201–209. Available from: http://www.neurology.org/content/74/3/201.abstract. 20042704 10.1212/WNL.0b013e3181cb3e25 PMC2809036

[33] Fjell AM, McEvoy L, Holland D, Dale AM, Walhovd KB, ADNI. Brain changes in older adults at very low risk for Alzheimer’s disease. J Neurosci. 2013 5;33(19):8237–8242. Available from: 10.1523/JNEUROSCI.5506-12.2013. 23658162 PMC4050197

[34] Desikan R, Cabral H, Hess C, Dillon W, Glastonbury C, Weiner M, et al Automated MRI measures identify individuals with mild cognitive impairment and Alzheimer’s disease. Brain. 2009;132(8):2048–2057. 10.1093/brain/awp123 19460794 PMC2714061

[35] Desikan RS, Cabral HJ, Settecase F, Hess CP, Dillon WP, Glastonbury CM, et al Automated MRI measures predict progression to Alzheimer’s disease. Neurobiol Aging. 2010 8;31(8):1364–1374. 10.1016/j.neurobiolaging.2010.04.023 20570399 PMC2902697

[36] Weiner MW, Veitch DP, Aisen PS, Beckett LA, Cairns NJ, Green RC, et al The Alzheimer’s Disease Neuroimaging Initiative: A review of papers published since its inception. Alzheimer’s and Dementia. 2012;8(1, Supplement):S1–S68. 10.1016/j.jalz.2011.09.172 22047634 PMC3329969

[37] Jack C, Bernstein M, Fox N. The Alzheimer’s Disease Neuroimaging Initiative (ADNI): MRI methods. J Magn Reson Imaging. 2008;27(4):685–691. 10.1002/jmri.21049 18302232 PMC2544629

[38] Dickerson BC, Bakkour A, Salat DH, Feczko E, Pacheco J, Greve DN, et al The cortical signature of Alzheimer’s disease: regionally specific cortical thinning relates to symptom severity in very mild to mild AD dementia and is detectable in asymptomatic amyloid-positive individuals. Cereb Cortex. 2009 3;19(3):497–510. Available from: 10.1093/cercor/bhn113. 18632739 PMC2638813

[39] Cho Y, Seong JK, Jeong Y, Shin SY, Initiative ADN. Individual subject classification for Alzheimer’s disease based on incremental learning using a spatial frequency representation of cortical thickness data. Neuroimage. 2012 2;59(3):2217–2230. Available from: 10.1016/j.neuroimage.2011.09.085. 22008371 PMC5849264

[40] Desikan R, Sigonne F, Fischl B, Quinn BT, Dickerson BC, Blacker D, et al An automated labeling system for subdividing the human cerebral cortex on MRI scans into gyral based regions of interest. NeuroImage. 2006;31(3):968–980. Available from: http://www.sciencedirect.com/science/article/pii/S1053811906000437. 16530430 10.1016/j.neuroimage.2006.01.021

[41] Fischl B. FreeSurfer. Neuroimage. 2012 1;Available from: 10.1016/j.neuroimage.2012.01.021. 22248573 PMC3685476

[42] Tosun D, Schuff N, Truran-Sacrey D, Shaw LM, Trojanowski JQ, Aisen P, et al Relations between brain tissue loss, CSF biomarkers, and the ApoE genetic profile: a longitudinal MRI study. Neurobiol Aging. 2010 8;31(8):1340–1354. 10.1016/j.neurobiolaging.2010.04.030 20570401 PMC2902689

[43] Bates D, Mächler M, Bolker B, Walker S. Fitting Linear Mixed-Effects Models Using lme4. Journal of Statistical Software. 2015;67(1):1–48. 10.18637/jss.v067.i01

[44] Höskuldsson A. PLS regression methods. Journal of Chemometrics. 1988;2(3):211–228. Available from: 10.1002/cem.1180020306.

[45] Wold S, Sjöström M, Eriksson L. PLS-regression: a basic tool of chemometrics. Chemometrics and Intelligent Laboratory Systems. 2001;58(2):109–130. {PLS} Methods. Available from: http://www.sciencedirect.com/science/article/pii/S0169743901001551.

[46] Dayal BS, MacGregor JF. Improved PLS algorithms. Journal of Chemometrics. 1997;11(1):73–85. Available from: http://onlinelibrary.wiley.com/doi/10.1002/(SICI)1099-128X(199701)11:1%3C73::AID-CEM435%3E3.0.CO;2-%23/abstract.

[47] Mevik BH, Wehrens R, Liland KH. pls: Partial Least Squares and Principal Component regression; 2013. R package version 2.4-3 Available from: http://CRAN.R-project.org/package=pls.

[48] Coffey CE, Lucke JF, Saxton JA, Ratcliff G, Unitas LJ, Billig B, et al Sex differences in brain aging: a quantitative magnetic resonance imaging study. Arch Neurol. 1998;55(2):169–79. Available from: http://www.ncbi.nlm.nih.gov/pubmed/9482358. 10.1001/archneur.55.2.169 9482358

[49] Xu J, Kobayashi S, Yamaguchi S, Iijima K, Okada K, Yamashita K. Gender effects on age-related changes in brain structure. AJNR Am J Neuroradiol. 2000;21(1):112–118. 10669234 PMC7976349

[50] Király A, Szabó N, Tóth E, Csete G, Faragó P, Kocsis K, et al Male brain ages faster: the age and gender dependence of subcortical volumes. Brain Imaging and Behavior. 2015;p. 1–10. Available from: 10.1007/s11682-015-9468-3. 26572143

[51] Toledo JB, Xie SX, Trojanowski JQ, Shaw LM. Longitudinal change in CSF Tau and ABeta biomarkers for up to 48 months in ADNI. Acta Neuropathologica. 2013;126(5):659–670. 10.1007/s00401-013-1151-4 23812320 PMC3875373

[52] De Meyer G, Shapiro F, Vanderstichele H, Vanmechelen E, Engelborghs S, De Deyn PP, et al Diagnosis-independent Alzheimer disease biomarker signature in cognitively normal elderly people. Arch Neurol. 2010 8;67(8):949–956. 10.1001/archneurol.2010.179 20697045 PMC2963067

[53] Chupin M, Gérardin E, Cuingnet R, Boutet C, Lemieux L, Lehéricy S, et al Fully automatic hippocampus segmentation and classification in Alzheimer’s disease and mild cognitive impairment applied on data from ADNI. Hippocampus. 2009 6;19(6):579–587. Available from: 10.1002/hipo.20626. 19437497 PMC2837195

[54] Das SR, Avants BB, Pluta J, Wang H, Suh JW, Weiner MW, et al Measuring longitudinal change in the hippocampal formation from in vivo high-resolution T2-weighted MRI. Neuroimage. 2012 4;60(2):1266–1279. Available from: 10.1016/j.neuroimage.2012.01.098. 22306801 PMC3667607

