# Supplementary material for: Early Prediction of Alzheimer’s Disease Using Null Longitudinal Model-Based Classifiers
Source: PLoS One. 2017 Jan 3;12(1):e0168011. doi: 10.1371/journal.pone.0168011 (PMC5207395; doi:10.1371/journal.pone.0168011)
Supplement: S1 Appendix — (PDF) [file pone.0168011.s001.pdf]

S1 Appendix: Brief Data and Methods Description.

ADNI diagnosis criteria

The general inclusion-exclusion criteria applied by ADNI to a baseline clinical assessment are: (1) Control normal subjects (CN) had MMSE scores between 24 and 30 (inclusive), a CDR of 0. They were non-depressed, non MCI, and non-demented; (2) Late MCI (LMCI) subjects had MMSE scores between 24 and 30 (inclusive), a memory complaint, had objective memory loss measured by education adjusted scores on Wechsler Memory Scale Logical Memory II, a CDR of 0.5, absence of significant levels of impairment in other cognitive domains, essentially preserved activities of daily living, and an absence of dementia, and (3) AD subjects had MMSE scores between 20 and 26 (inclusive), CDR of 0.5 or 1.0, and met "National Institute of Neurological and Communicative Diseases and Stroke Alzheimer's Disease and Related Disorders Association" (NINCDS/ADRDA) criteria for probable AD.

Sociodemographic and clinical ADNI data

APOE-ϵ4 carrier state include the several states: 0: non-carrier, 1: single copy carrier, 2: two copies carrier); all of these features were documented in the screen visit of ADNI participants. These features have also been considered in other dementia studies based on ADNI database.

CDRGLOBAL indicate severity of dementia (0: no dementia, 0.5: very mild dementia, 1: mild dementia, 2: moderate dementia, 3: severe dementia); and it is obtained by using an algorithm that weights memory more heavily than the other remaining five categories (orientation, judgment and problem solving, community affairs/involvement, home life and hobbies, and personal care).

MMSE and CDGLOBAL are available for each participant visit and are the basis of ADNI for baseline clinical assessment.

PLSR modelling

By definition, after observing n data samples from each block of variables, PLSR decomposes the $n \times N$ matrix of zero-mean predictors variables Y_{nv} and the $n \times M$ matrix of zero-mean responses variables Y_v into the form shown in Eq (1).

$$\begin{aligned} Y_{nv} = &\quad TP^T + E \\ Y_v = &\quad UQ^T + F \end{aligned} \tag{1}$$

where $Y_{nv} \subset R^N$ and $Y_v \subset R^M$ represent the y_0 values of vr and qvr ROIs, respectively. T and U are $n \times p$ matrices that are the p extracted score vectors (projections, components, latent vectors) of Y_{nv} and Y_v, respectively. The $N \times p$ matrix P and the $M \times p$ matrix Q represent matrices of loadings; and the $n \times N$ matrix E and the $n \times M$ matrix F are the matrices of residuals (or error matrices), assumed to be independent and identically distributed random normal variables. The decompositions of Y_{nv} and Y_v are made to maximize the covariance between T and U.

Final LME formulation

LME modelling for each ROI was applied separately in men and women by assuming different random intercepts (at baseline response) for each subject. Also, the effect of *age* (βa) and *educ* (βe) was assumed the same for all subjects. The y-intercept varies between subjects, but it is the same for all subjects' observations. Eq (2) describes the LME formulation used to model the change of every MRI biomarker.

$$
\begin{aligned}
y_{ij}^r = \;& \beta_1^r Intercept_{ij} + \beta_a^r age_{ij} \\
& + \beta_e^r educ_{ij} + \alpha_{i1} Intercept_{ij} + \varepsilon_{ij}
\end{aligned} \tag{2}
$$

where $i = 1, ..., n$ subjects; n is the number of normal-HC$_{\text{csf}}$ subjects (n=46); $j = 1, ..., n_i$; n_i is equal to the number of observations per subject; $r = 1, ..., nr$ biomarkers, nr=166 ROIs. y_{ij}^r is the value of the r^{th}ROI for the jth of n_i observations in the subject i. The coefficients β_1^r, β_a^r and β_e^r represent a p×1 vector of unknown fixed effect parameters of ROI r, being p the number of fixed effects including the intercept. These β's vary between ROIs, but they are fixed for all subject's observations. $Intercept_{ij}$, age_{ij} and $educ_{ij}$ are the set of fixed-effects covariates or regressors for the jth response on the ith subject. $Intercept_{ij}$ regressor is constant and equal to 1. α_{i1} is the random effects coefficient for the ith subject and it varies between subjects. ϵ_{ij} is the error for the jth observation in subject i.

By reorganizing terms, the formulation of mixed-effects model defined in Eq (2) can be written as Eq (3).

$$
\begin{aligned}
y_{ij}^r = \;& (\beta_1 Intercept_{ij} + \alpha_{i1} Intercept_{ij}) + \beta_a age_{ij} \\
& + \beta_e educ_{ij} + \epsilon_{ij}
\end{aligned} \tag{3}
$$

where the summation $(\beta_1 Intercept_{ij} + \alpha_{i1} Intercept_{ij})$ represents the y_0 (y-intercept value at basal stage), see figure in S1 Fig. In Eq (4) is represented the matrix and vector notation of Eq (3),

$$
y_{ij}^r = y_{0ij} + X_{ij}\beta^r + \epsilon_{ij}^r \tag{4}
$$

where β^r =(β_a,β_e)'. X_{ij} is the design matrix with the values of age_{ij} and $educ_{ij}$ regressors (without the constant term).

Application of proposed method in a hypothetical example

Figure in S1 Fig illustrates a hypothetical example of how we have used the LME and PLSR approaches to infer the ROI values at basal stage and over time; and then to infer the residuals. The figure shows an example of LME-based trajectories for hypothetical variant and quasi-variant ROIs fitted on healthy elderly data. In each plot, P_1, P_2 and P_3 represent hypothetical observations of each ROI y for two subjects at three different ages (a_1, a_2 and a_3). The first subject is assumed as HC and the second subject is assumed as AD, and it is

assumed that neither subject was used to build the models. The black lines represent the healthy population regression line calculated for each ROI, where $\hat{y}_0$ represents the vertical y-intercept value of healthy population. The blue and red lines represent the individual regression lines estimated for both subjects by assuming both as healthy; and the points $\hat{P}_1$, $\hat{P}_2$ and $\hat{P}_3$ represent the inferred $\hat{y}$'s for the three ages. Observe that, $\hat{y}_{HC_0}$ and $\hat{y}_{AD_0}$ are the subject-specific y-intercepts estimated for HC and AD subjects, respectively. For both cases, $\hat{y}_{HC_0}$ and $\hat{y}_{AD_0}$ of vr ROI are inferred from the $\hat{y}_{HC_0}$ and $\hat{y}_{AD_0}$ of qvr ROI using the PLSR model (as described above). The slope β_a is the rate change of the standard deviation of ROI per unit of age; and this slope is the same for both estimated individual regression lines. ϵ_{HC1}, ϵ_{HC2}, ϵ_{HC3}, ϵ_{AD1}, ϵ_{AD2} and ϵ_{AD3} are the residuals of each observation with respect to the estimated individual regression lines, which are computed in general way as $y - \hat{y}$. Here, the figure shows that AD residuals are greater than HC residuals because this subject is possibly affected by further neurodegeneration.
