# Supplementary material for: Early Prediction of Alzheimer’s Disease Using Null Longitudinal Model-Based Classifiers
Source: PLoS One. 2017 Jan 3;12(1):e0168011. doi: 10.1371/journal.pone.0168011 (PMC5207395; doi:10.1371/journal.pone.0168011)
Supplement: S1 Fig — HC and AD are hypothetical subjects. P1, P2 and P3 are observations of each ROI y at three different ages (a1, a2 and a3). Black lines, healthy population regression line calculated from LME. y^0, vertical y-intercept value of healthy population. Blue and red lines, individual regression lines estimated by assuming both as healthful. Points P^1, P^2 and P^3, inferred y^’s for the three ages.y^HC0 and y^AD0, the subject-specific y-intercepts estimated for HC and AD subjects, respectively. y^HC0 and y^AD0 of vr ROI are inferred from the y^HC0 and y^AD0 of qvr ROI through PLSR model. βa, slope or rate change of standard deviation of ROI per unit of age. ϵHC1, ϵHC2, ϵHC3, ϵAD1, ϵAD2 and ϵAD3, the residuals of each observation with respect to the estimated individual regression lines. (PDF) [file pone.0168011.s002.pdf]

S1 Fig.

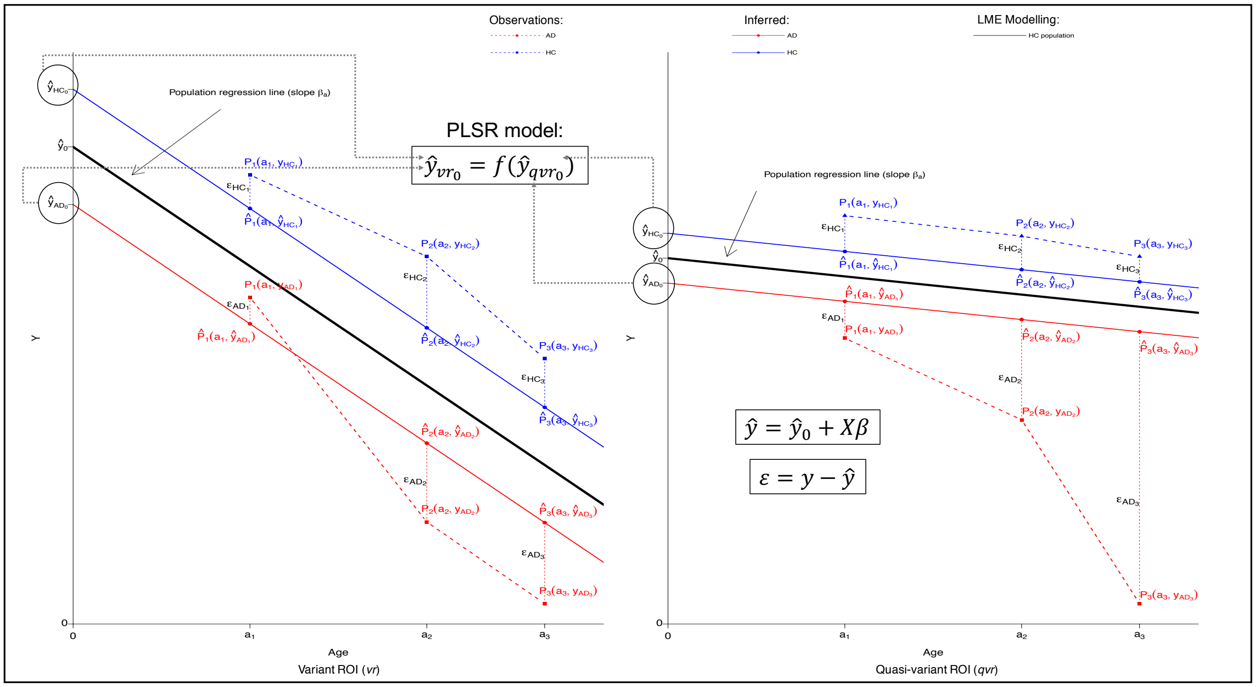

Fig 1. Example of LME modelling for hypothetical variant (vr) and quasi-variant(qvr) ROIs.

HC and AD are hypothetical subjects. P_1, P_2 and P_3 are observations of each ROI y at three different ages (a_1, a_2 and a_3). Black lines, healthy population regression line calculated from LME. $\hat{y}_0$, vertical y-intercept value of healthy population. Blue and red lines, individual regression lines estimated by assuming both as healthy. Points $\hat{P}_1$, $\hat{P}_2$ and $\hat{P}_3$, inferred $\hat{y}$'s for the three ages.$\hat{y}_{HC_0}$ and $\hat{y}_{AD_0}$, the subject-specific y-intercepts estimated for HC and AD subjects, respectively. $\hat{y}_{HC_0}$ and $\hat{y}_{AD_0}$ of vr ROI are inferred from the $\hat{y}_{HC_0}$ and $\hat{y}_{AD_0}$ of qvr ROI through PLSR model. β_a, slope or rate change of the standard deviation of ROI per unit of age. ϵ_{HC1}, ϵ_{HC2}, ϵ_{HC3}, ϵ_{AD1}, ϵ_{AD2} and ϵ_{AD3}, the residuals of each observation with respect to the estimated individual regression lines.
