# Supplementary material for: Early Prediction of Alzheimer’s Disease Using Null Longitudinal Model-Based Classifiers
Source: PLoS One. 2017 Jan 3;12(1):e0168011. doi: 10.1371/journal.pone.0168011 (PMC5207395; doi:10.1371/journal.pone.0168011)
Supplement: S2 Fig — (a) CSF-Aβ. (b) CSF-τ. △: 8 of 113 CN subjects were converted to MCI and 1 CN subject was converted to AD. ○: 88 of 94 LMCI were converted to AD and 5 were re-converted to HC at follow-up visits. Dotted vertical lines within each diagnosis are the determined cutoff concentrations. Figure shows us that several subjects with abnormal CSF profile classified as CN by ADNI at baseline clinical assessment were converted to MCI or AD (triangle dots) along time. Likewise, some subjects classified like ‘LMCI’ by ADNI were converted to AD later (open circles). (PDF) [file pone.0168011.s003.pdf]

S2 Fig. Distribution of CSF-based biomarkers at follow-up 84 months.

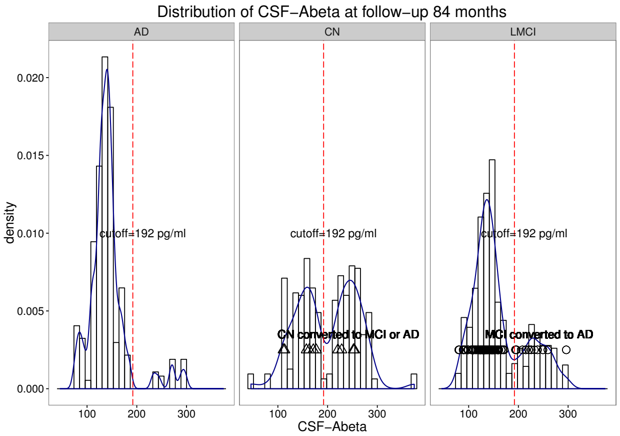

(a)

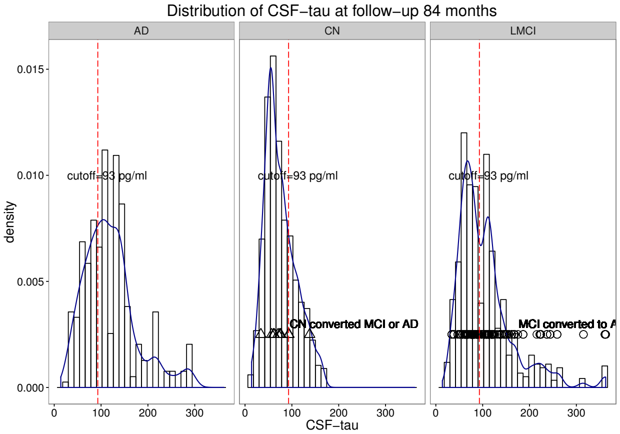

(b)

Fig 2. Distribution of CSF-based biomarkers at follow-up 84 months.

(a) CSF-Aβ. (b) CSF-τ.
$\triangle$: 8 of 113 CN subjects were converted to MCI and 1 CN subject was converted to AD. $\bigcirc$: 88 of 94 LMCI were converted to AD and 5 were re-converted to HC at follow-up visits. Dotted vertical lines within each diagnosis are the determined cutoff concentrations. Figure shows us that several subjects with abnormal CSF profile classified as CN by ADNI at baseline clinical assessment were converted to MCI or AD (triangle dots) along time. Likewise, some subjects classified like 'LMCI' by ADNI were converted to AD later (open circles).
