# Supplementary material for: Early Prediction of Alzheimer’s Disease Using Null Longitudinal Model-Based Classifiers
Source: PLoS One. 2017 Jan 3;12(1):e0168011. doi: 10.1371/journal.pone.0168011 (PMC5207395; doi:10.1371/journal.pone.0168011)
Supplement: S3 Fig — Dots represent the last CSF biomarker measured for subjects available at April, 2015. Vertical and horizontal dashed lines split normal CSF-profile from abnormal profile. Null models for characterization of healthy brain structures were built from samples labelled with blue dots. (PDF) [file pone.0168011.s004.pdf]

S3 Fig. CSF-Aβ vs. CSF-τ concentration available at last subjects' observations.

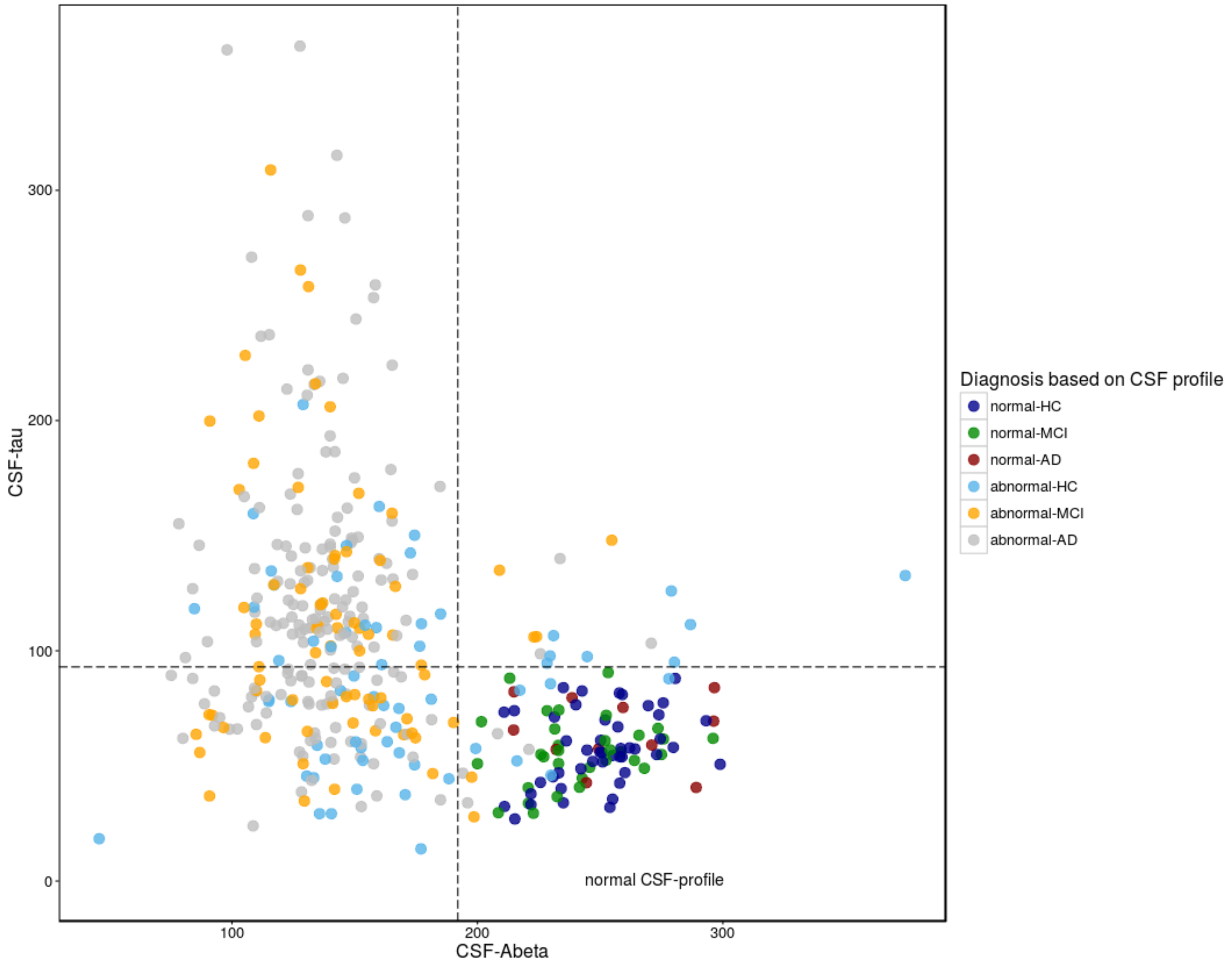

Fig 3. CSF-Aβ vs. CSF-τ concentration available at last subject" observations.

Dots represent the last CSF biomarker measured for subjects available at April, 2015. Vertical and horizontal dashed lines split normal CSF-profile from abnormal profile. Null models for characterization of healthy brain structures were built from samples labelled with blue dots.
