# Supplementary material for: Early Prediction of Alzheimer’s Disease Using Null Longitudinal Model-Based Classifiers
Source: PLoS One. 2017 Jan 3;12(1):e0168011. doi: 10.1371/journal.pone.0168011 (PMC5207395; doi:10.1371/journal.pone.0168011)
Supplement: S4 Fig — High between-subject variability is evident, e.g., by comparing subject 099_S_0533 with subject 133_S_0488. Likewise, there is a strong indication of gender effect over hippocampal volume, female volumes are less than the male ones. Note that we standarized every MRI-based biomarker to have zero mean and unit variance. (PDF) [file pone.0168011.s005.pdf]

S4 Fig. Boxplot of trajectory of left hippocampal volume for normal-HC$_{\text{csf}}$ subjects.

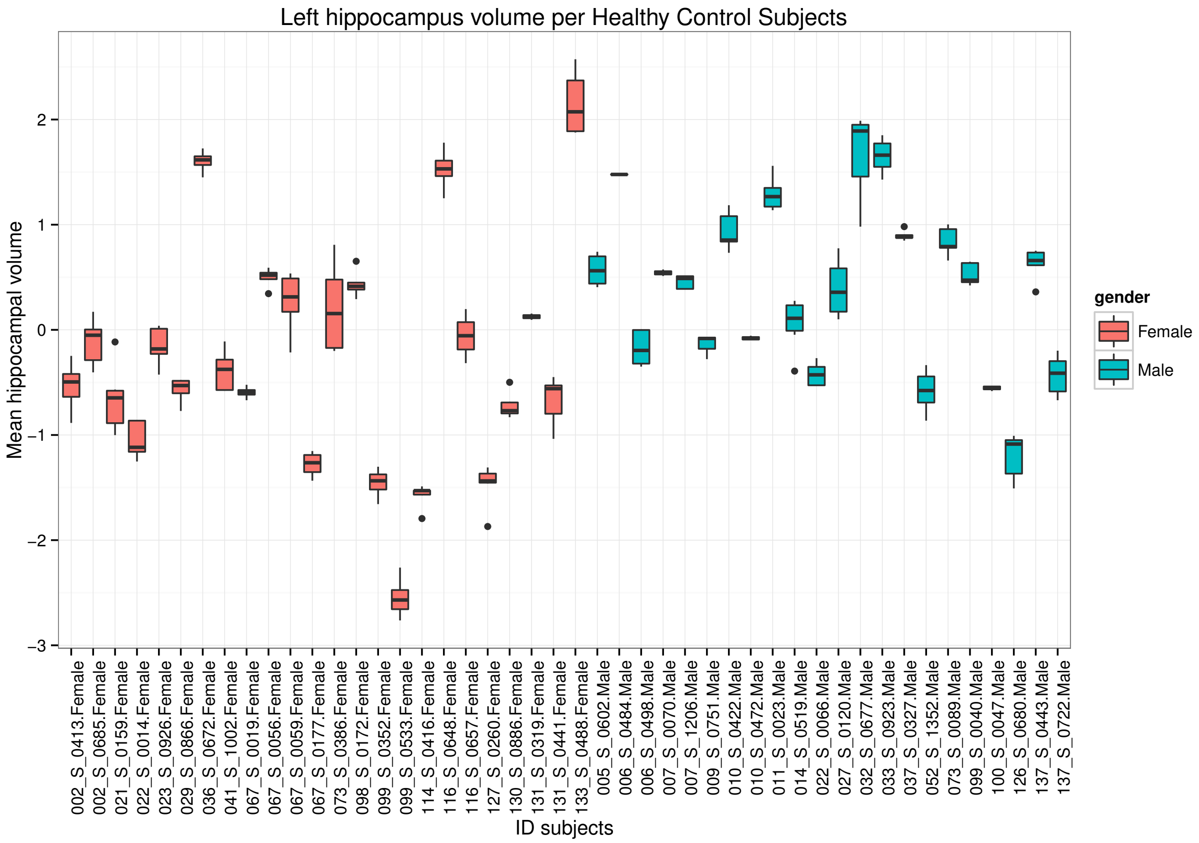

Fig 4. Boxplot of trajectory of left hippocampal volume for normal-HC$_{\text{csf}}$ subjects.

High between-subject variability is evident, e.g., by comparing subject 099_S_0533 with subject 133_S_0488. Likewise, there is a strong indication of gender effect over hippocampal volume, female volumes are less than the man ones. Note that we standarized every MRI-based biomarker to have zero mean and unit variance.
