# Supplementary material for: Early Prediction of Alzheimer’s Disease Using Null Longitudinal Model-Based Classifiers
Source: PLoS One. 2017 Jan 3;12(1):e0168011. doi: 10.1371/journal.pone.0168011 (PMC5207395; doi:10.1371/journal.pone.0168011)
Supplement: S5 Fig — (a) Left hippocampal volume classified as variant (vr) ROI; where the slope of trajectories is not close to zero. (b) Left caudate volume classified as quasi-variant (qvr) ROI; where the slope of trajectories is close to zero. Note that for both regions, the y-intercept values vary between subjects, but the slope value of each ROI is the same for all subjects. (PDF) [file pone.0168011.s006.pdf]

S5 Fig. Examples of variant and quasi-variant ROIs for normal-HC$_{csf}$ subjects stratified by gender.

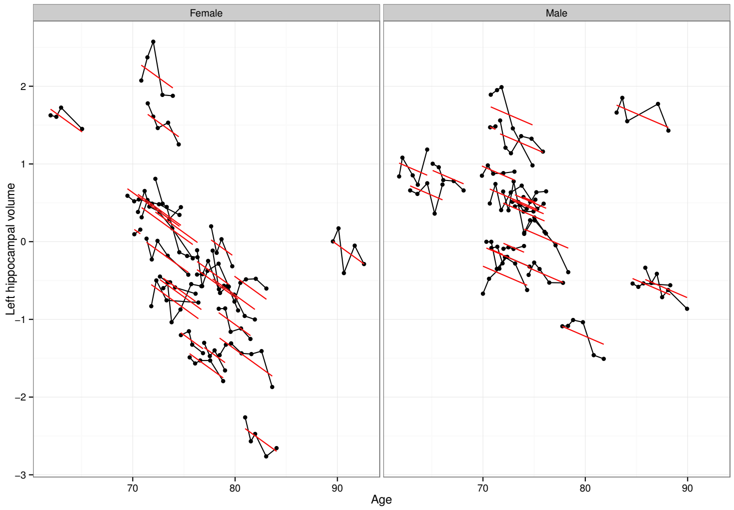

(a)

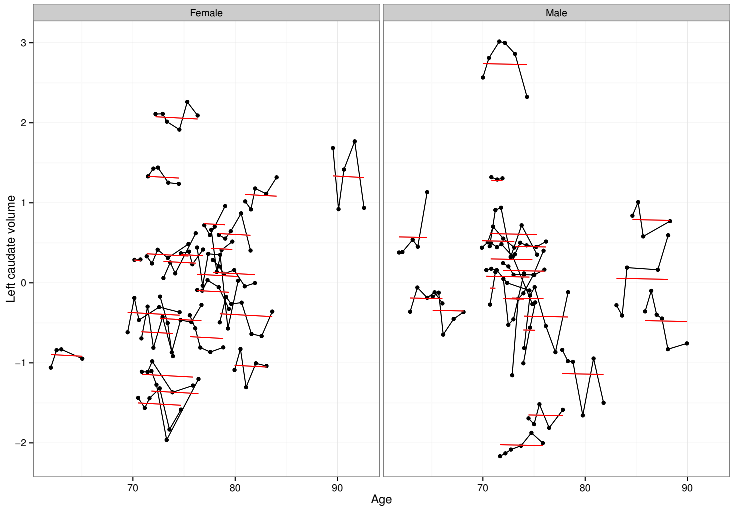

(b)

Fig 5. Examples of variant and quasi-variant ROIs per normal-HC$_{csf}$ subjects stratified by gender.

(a) Left hippocampal volume classified as variant (vr) ROI; where the slope of trajectories is not close to zero. (b) Left caudate volume classified as quasi-variant (qvr) ROI; where the slope of trajectories is close to zero. Note that for both regions, the y-intercept values vary between subjects, but the slope value of each ROI is the same for all subjects.
