# Supplementary material for: Early Prediction of Alzheimer’s Disease Using Null Longitudinal Model-Based Classifiers
Source: PLoS One. 2017 Jan 3;12(1):e0168011. doi: 10.1371/journal.pone.0168011 (PMC5207395; doi:10.1371/journal.pone.0168011)
Supplement: S6 Fig — (a) Males. (b) Females. Biomarkers are coloured according their change type: blue for vr regions and red for the qvr ones. The size of biomarkers with significant P-value ≤ 0.05 are bigger than the not significant. Dotted vertical lines separate the increased biomarkers (positive beta values) from the reduced ones (negative beta values). ROIs are represented according to their location into brain hemisphere (lh:left, rh: right or bilateral). SV: Subcortical Volume, CV: Cortical volume, SA: Surface Area. lh: left hemisphere. rh: right hemisphere. (PDF) [file pone.0168011.s007.pdf]

# S6 Fig. Characterization of ageing-based variant ($vr$) and quasi-variant ($qvr$) ROIs.

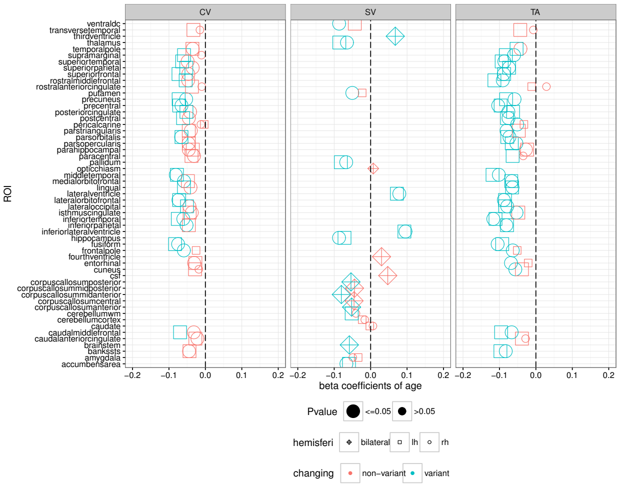


(a)

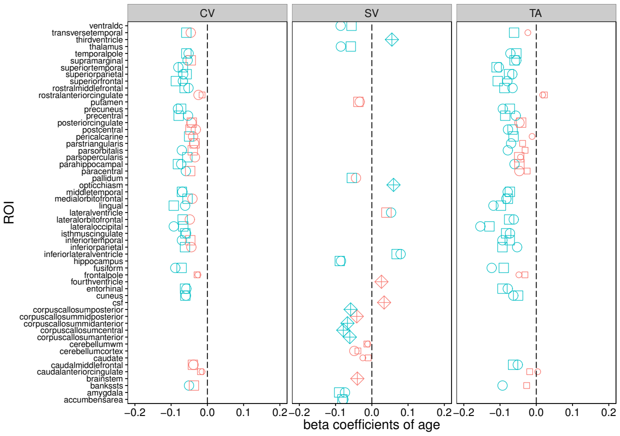


(b)

**Fig 6. Characterization of Ageing-based variant ($vr$) and quasi-variant ($qvr$) ROIs.**

(a) Males. (b) Females. Biomarkers are coloured according their change type: blue for $vr$ regions and red for the $qvr$ ones. The size of biomarkers with significant $P$-value $\leq 0.05$ are bigger than the not significant. Dotted vertical lines separate the increased biomarkers (positive beta values) from the reduced ones (negative beta values). ROIs are represented according to their location into brain hemisphere (lh:left, rh: right or bilateral). SV: Subcortical Volume, CV: Cortical volume, SA: Surface Area. lh: left hemisphere. rh: right hemisphere.
