# Supplementary material for: Early Prediction of Alzheimer’s Disease Using Null Longitudinal Model-Based Classifiers
Source: PLoS One. 2017 Jan 3;12(1):e0168011. doi: 10.1371/journal.pone.0168011 (PMC5207395; doi:10.1371/journal.pone.0168011)
Supplement: S1 Table — (PDF) [file pone.0168011.s008.pdf]

S1 Table. Statistical descriptors of studied ADNI cohort at baseline.

Table 1. Statistical descriptors of studied ADNI cohort at baseline.

	Female<br>$N = 316$	Male<br>$N = 431$
Age_{bl}	$_{71.1}$ 75.3 $_{79.9}$ (74.8± 6.8)	$_{71.2}$ 75.6 $_{79.9}$ (75.4± 6.7)
Years of education	$_{0.65}$ 0.75 $_{0.85}$ (0.75±0.14)	$_{0.70}$ 0.80 $_{0.90}$ (0.80±0.15)
Ethnicity		
Hisp/Latino	2.2% $\frac{7}{316}$	1.9% $\frac{8}{431}$
Not Hisp/Latino	96.5% $\frac{305}{316}$	98.1% $\frac{423}{431}$
Unknown	1.3% $\frac{4}{316}$	0.0% $\frac{0}{431}$
Race		
Am Indian/Alaskan	0.32% $\frac{1}{316}$	0.00% $\frac{0}{431}$
Asian	1.58% $\frac{5}{316}$	2.09% $\frac{9}{431}$
Black	6.33% $\frac{20}{316}$	3.25% $\frac{14}{431}$
More than one	0.32% $\frac{1}{316}$	0.23% $\frac{1}{431}$
White	91.46% $\frac{289}{316}$	94.43% $\frac{407}{431}$
Marital State		
Divorced	10.44% $\frac{33}{316}$	3.25% $\frac{14}{431}$
Married	61.08% $\frac{193}{316}$	89.10% $\frac{384}{431}$
Never married	4.43% $\frac{14}{316}$	2.09% $\frac{9}{431}$
Unknown	0.00% $\frac{0}{316}$	0.23% $\frac{1}{431}$
Widowed	24.05% $\frac{76}{316}$	5.34% $\frac{23}{431}$
APOE-ϵ4 *		
0	52% $\frac{164}{316}$	50% $\frac{215}{431}$
1	37% $\frac{116}{316}$	39% $\frac{167}{431}$
2	11% $\frac{36}{316}$	11% $\frac{49}{431}$
MMSE	$_{25.0}$ 27.0 $_{29.0}$ (26.8± 2.7)	$_{25.0}$ 27.0 $_{29.0}$ (26.8± 2.5)
CDRGLOBAL		
0	32.6% $\frac{103}{316}$	26.0% $\frac{112}{431}$
0.5	54.4% $\frac{172}{316}$	65.7% $\frac{283}{431}$
1	13.0% $\frac{41}{316}$	8.3% $\frac{36}{431}$
dx_{bl}		
AD	25% $\frac{80}{316}$	20% $\frac{86}{431}$
CN	33% $\frac{103}{316}$	26% $\frac{112}{431}$
LMCI	42% $\frac{133}{316}$	54% $\frac{233}{431}$
Availability of CSF data **		
Yes	52% $\frac{164}{316}$	55% $\frac{236}{431}$
Not	48% $\frac{152}{316}$	45% $\frac{195}{431}$

$_a\, b\, _c$ represent the lower quartile a, the median b, and the upper quartile c for continuous variables. $x \pm s$ represents $\bar{X} \pm 1$ SD.N is the number of non–missing values observations. Numbers after percents are frequencies. AD: dementia due to Alzheimer‘s Disease; CN: Control Normal and LMCI: Late Mild Cognitive Impairment.

* Number of copies of allele.

** Availability of both CSF-Aβ and CSF-$\tau$was data at time of study (April, 2015).
