# Supplementary material for: Early Prediction of Alzheimer’s Disease Using Null Longitudinal Model-Based Classifiers
Source: PLoS One. 2017 Jan 3;12(1):e0168011. doi: 10.1371/journal.pone.0168011 (PMC5207395; doi:10.1371/journal.pone.0168011)
Supplement: S2 Table — (PDF) [file pone.0168011.s009.pdf]

S2 Table. Statistical descriptors of males used to build the SVM.

Table 2. Statistical descriptors of males used to build the SVM.

	N	55–59	60–64	65–69	70–74	75–79	80–85	85–89	90+
		$N = 23$	$N = 86$	$N = 152$	$N = 459$	$N = 554$	$N = 420$	$N = 209$	$N = 5$
age	1908	57.71 58.67 59.37 (58.46± 1.19)	62.38 63.47 64.28 (63.19± 1.32)	66.26 67.90 69.17 (67.73± 1.56)	71.81 73.03 74.02 (72.88± 1.35)	76.28 77.41 78.60 (77.44± 1.41)	80.95 82.18 83.61 (82.29± 1.49)	85.83 86.78 87.82 (86.94± 1.33)	90.21 90.35 90.61 (90.40± 0.26)
dx_{csf}	1003								
normal-MCI_{csf}		25.0% $\frac{3}{12}$	30.9% $\frac{13}{42}$	14.3% $\frac{12}{84}$	9.4% $\frac{21}{224}$	4.1% $\frac{13}{315}$	10.2% $\frac{21}{205}$	17.2% $\frac{20}{116}$	0.0% $\frac{0}{5}$
normal-AD_{csf}		0.0% $\frac{0}{12}$	0.0% $\frac{0}{42}$	0.0% $\frac{0}{84}$	4.9% $\frac{11}{224}$	3.2% $\frac{10}{315}$	3.4% $\frac{7}{205}$	5.2% $\frac{6}{116}$	0.0% $\frac{0}{5}$
abnormal-HC_{csf}		0.0% $\frac{0}{12}$	9.5% $\frac{4}{42}$	7.1% $\frac{6}{84}$	21.9% $\frac{49}{224}$	22.9% $\frac{72}{315}$	11.7% $\frac{24}{205}$	14.7% $\frac{17}{116}$	40.0% $\frac{2}{5}$
abnormal-MCI_{csf}		0.0% $\frac{0}{12}$	2.4% $\frac{1}{42}$	23.8% $\frac{20}{84}$	21.0% $\frac{47}{224}$	30.8% $\frac{97}{315}$	24.4% $\frac{50}{205}$	19.0% $\frac{22}{116}$	0.0% $\frac{0}{5}$
abnormal-AD_{csf}		75.0% $\frac{9}{12}$	57.1% $\frac{24}{42}$	54.8% $\frac{46}{84}$	42.9% $\frac{96}{224}$	39.0% $\frac{123}{315}$	50.2% $\frac{103}{205}$	44.0% $\frac{51}{116}$	60.0% $\frac{3}{5}$
dx_{last}	1908								
AD		60.9% $\frac{14}{23}$	43.0% $\frac{37}{86}$	59.2% $\frac{90}{152}$	44.9% $\frac{206}{459}$	39.9% $\frac{221}{554}$	46.2% $\frac{194}{420}$	41.6% $\frac{87}{209}$	60.0% $\frac{3}{5}$
HC		4.3% $\frac{1}{23}$	12.8% $\frac{11}{86}$	9.2% $\frac{14}{152}$	27.2% $\frac{125}{459}$	29.8% $\frac{165}{554}$	16.2% $\frac{68}{420}$	20.1% $\frac{42}{209}$	40.0% $\frac{2}{5}$
MCI		34.8% $\frac{8}{23}$	44.2% $\frac{38}{86}$	31.6% $\frac{48}{152}$	27.9% $\frac{128}{459}$	30.3% $\frac{168}{554}$	37.6% $\frac{158}{420}$	38.3% $\frac{80}{209}$	0.0% $\frac{0}{5}$
dx_{age}	1908								
AD		30.4% $\frac{7}{23}$	33.7% $\frac{29}{86}$	34.9% $\frac{53}{152}$	24.6% $\frac{113}{459}$	26.9% $\frac{149}{554}$	29.1% $\frac{122}{420}$	27.3% $\frac{57}{209}$	60.0% $\frac{3}{5}$
HC		4.3% $\frac{1}{23}$	11.6% $\frac{10}{86}$	9.2% $\frac{14}{152}$	27.2% $\frac{125}{459}$	30.9% $\frac{171}{554}$	19.3% $\frac{81}{420}$	21.5% $\frac{45}{209}$	40.0% $\frac{2}{5}$
MCI		65.2% $\frac{15}{23}$	54.6% $\frac{47}{86}$	55.9% $\frac{85}{152}$	48.1% $\frac{221}{459}$	42.2% $\frac{234}{554}$	51.7% $\frac{217}{420}$	51.2% $\frac{107}{209}$	0.0% $\frac{0}{5}$
APOE-ϵ4	1908								
0		69.6% $\frac{16}{23}$	54.6% $\frac{47}{86}$	28.3% $\frac{43}{152}$	47.7% $\frac{219}{459}$	48.6% $\frac{269}{554}$	53.1% $\frac{223}{420}$	56.9% $\frac{119}{209}$	40.0% $\frac{2}{5}$
1		13.0% $\frac{3}{23}$	38.4% $\frac{33}{86}$	44.1% $\frac{67}{152}$	37.7% $\frac{173}{459}$	41.2% $\frac{228}{554}$	40.0% $\frac{168}{420}$	38.8% $\frac{81}{209}$	40.0% $\frac{2}{5}$
2		17.4% $\frac{4}{23}$	7.0% $\frac{6}{86}$	27.6% $\frac{42}{152}$	14.6% $\frac{67}{459}$	10.3% $\frac{57}{554}$	6.9% $\frac{29}{420}$	4.3% $\frac{9}{209}$	20.0% $\frac{1}{5}$
MMSE	1906	25.0 26.0 28.5 (26.7± 2.3)	24.0 27.0 29.0 (26.0± 3.5)	23.0 26.0 29.0 (25.4± 4.2)	25.0 27.0 29.0 (26.4± 3.7)	25.0 28.0 29.0 (26.3± 3.9)	24.0 27.0 29.0 (26.2± 3.6)	24.0 27.0 28.0 (25.8± 3.4)	26.0 27.0 27.0 (25.2± 4.7)
CDRGLOBAL	1899	1.8 2.5 3.2 (2.7±1.3)	0.5 1.2 4.0 (2.2±2.0)	1.0 2.0 4.0 (2.6±2.2)	0.0 1.5 3.0 (2.1±2.2)	0.0 1.5 3.5 (2.3±2.6)	0.5 1.5 3.5 (2.3±2.3)	1.0 2.0 3.5 (2.7±2.9)	0.0 4.0 5.5 (3.7±3.8)

$_a\, b\, _c$ represent the lower quartile a, the median b, and the upper quartile c for continuous variables. $x \pm s$ represents $\bar{X} \pm 1$ SD. N: number of samples available at each age group, it being possible to have more of one observation per subject.
