# Supplementary material for: Early Prediction of Alzheimer’s Disease Using Null Longitudinal Model-Based Classifiers
Source: PLoS One. 2017 Jan 3;12(1):e0168011. doi: 10.1371/journal.pone.0168011 (PMC5207395; doi:10.1371/journal.pone.0168011)
Supplement: S3 Table — (PDF) [file pone.0168011.s010.pdf]

S3 Table. Statistical descriptors of females used to build the SVM.

Table 3. Statistical descriptors of females used to build the SVM

	N	55–59	60–64	65–69	70–74	75–79	80–85	85–89	90+
		$N = 37$	$N = 62$	$N = 117$	$N = 338$	$N = 355$	$N = 287$	$N = 101$	$N = 5$
age	1302	56.6 57.5 58.5 (57.6± 1.3)	61.5 62.6 64.0 (62.6± 1.4)	66.4 68.0 69.2 (67.8± 1.5)	71.6 72.8 73.9 (72.7± 1.4)	76.3 77.5 78.6 (77.5± 1.4)	81.0 82.2 83.5 (82.3± 1.4)	85.6 86.5 87.4 (86.6± 1.2)	90.9 91.4 92.8 (91.6± 1.2)
dx_{csf}	640								
normal-MCI_{csf}		0.0% $\frac{0}{29}$	29.7% $\frac{11}{37}$	16.0% $\frac{12}{75}$	3.0% $\frac{5}{167}$	2.5% $\frac{4}{161}$	12.1% $\frac{16}{132}$	10.5% $\frac{4}{38}$	0.0% $\frac{0}{1}$
normal-AD_{csf}		0.0% $\frac{0}{29}$	0.0% $\frac{0}{37}$	0.0% $\frac{0}{75}$	0.0% $\frac{0}{167}$	2.5% $\frac{4}{161}$	3.8% $\frac{5}{132}$	15.8% $\frac{6}{38}$	100.0% $\frac{1}{1}$
abnormal-HC_{csf}		0.0% $\frac{0}{29}$	0.0% $\frac{0}{37}$	1.3% $\frac{1}{75}$	22.8% $\frac{38}{167}$	33.5% $\frac{54}{161}$	25.0% $\frac{33}{132}$	26.3% $\frac{10}{38}$	0.0% $\frac{0}{1}$
abnormal-MCI_{csf}		17.2% $\frac{5}{29}$	54.0% $\frac{20}{37}$	21.3% $\frac{16}{75}$	21.6% $\frac{36}{167}$	18.6% $\frac{30}{161}$	17.4% $\frac{23}{132}$	0.0% $\frac{0}{38}$	0.0% $\frac{0}{1}$
abnormal-AD_{csf}		82.8% $\frac{24}{29}$	16.2% $\frac{6}{37}$	61.3% $\frac{46}{75}$	52.7% $\frac{88}{167}$	42.9% $\frac{69}{161}$	41.7% $\frac{55}{132}$	47.4% $\frac{18}{38}$	0.0% $\frac{0}{1}$
dx_{last}	1302								
AD		81.1% $\frac{30}{37}$	27.4% $\frac{17}{62}$	64.1% $\frac{75}{117}$	49.1% $\frac{166}{338}$	38.0% $\frac{135}{355}$	45.6% $\frac{131}{287}$	51.5% $\frac{52}{101}$	80.0% $\frac{4}{5}$
HC		0.0% $\frac{0}{37}$	0.0% $\frac{0}{62}$	5.1% $\frac{6}{117}$	30.8% $\frac{104}{338}$	34.1% $\frac{121}{355}$	24.7% $\frac{71}{287}$	28.7% $\frac{29}{101}$	20.0% $\frac{1}{5}$
MCI		18.9% $\frac{7}{37}$	72.6% $\frac{45}{62}$	30.8% $\frac{36}{117}$	20.1% $\frac{68}{338}$	27.9% $\frac{99}{355}$	29.6% $\frac{85}{287}$	19.8% $\frac{20}{101}$	0.0% $\frac{0}{5}$
dx_{age}	1302								
AD		62.16% $\frac{23}{37}$	17.74% $\frac{11}{62}$	35.90% $\frac{42}{117}$	36.39% $\frac{123}{338}$	27.04% $\frac{96}{355}$	29.97% $\frac{86}{287}$	44.55% $\frac{45}{101}$	80.00% $\frac{4}{5}$
HC		0.00% $\frac{0}{37}$	0.00% $\frac{0}{62}$	4.27% $\frac{5}{117}$	33.14% $\frac{112}{338}$	38.87% $\frac{138}{355}$	28.22% $\frac{81}{287}$	29.70% $\frac{30}{101}$	20.00% $\frac{1}{5}$
MCI		37.84% $\frac{14}{37}$	82.26% $\frac{51}{62}$	59.83% $\frac{70}{117}$	30.47% $\frac{103}{338}$	34.08% $\frac{121}{355}$	41.81% $\frac{120}{287}$	24.75% $\frac{25}{101}$	0.00% $\frac{0}{5}$
NL to Dementia		0.00% $\frac{0}{37}$	0.00% $\frac{0}{62}$	0.00% $\frac{0}{117}$	0.00% $\frac{0}{338}$	0.00% $\frac{0}{355}$	0.00% $\frac{0}{287}$	0.99% $\frac{1}{101}$	0.00-% $\frac{0}{5}$
APOE-ϵ4	1302								
0		59.5% $\frac{22}{37}$	43.5% $\frac{27}{62}$	17.1% $\frac{20}{117}$	35.8% $\frac{121}{338}$	51.0% $\frac{181}{355}$	62.0% $\frac{178}{287}$	81.2% $\frac{82}{101}$	100.0% $\frac{5}{5}$
1		13.5% $\frac{5}{37}$	38.7% $\frac{24}{62}$	35.9% $\frac{42}{117}$	49.7% $\frac{168}{338}$	43.9% $\frac{156}{355}$	34.8% $\frac{100}{287}$	15.8% $\frac{16}{101}$	0.0% $\frac{0}{5}$
2		27.0% $\frac{10}{37}$	17.7% $\frac{11}{62}$	47.0% $\frac{55}{117}$	14.5% $\frac{49}{338}$	5.1% $\frac{18}{355}$	3.1% $\frac{9}{287}$	3.0% $\frac{3}{101}$	0.0% $\frac{0}{5}$
MMSE	1297	20.0 23.0 25.0 (22.2± 4.6)	25.2 28.0 28.0 (26.5± 3.5)	24.0 26.0 28.0 (25.8± 3.0)	24.0 27.0 29.0 (25.6± 4.8)	24.0 28.0 29.0 (26.2± 4.5)	23.5 27.0 29.0 (26.1± 3.5)	21.0 26.0 29.0 (24.9± 4.3)	24.0 25.5 26.8 (25.2± 3.3)
CDRGLOBAL	1289	3.0 4.0 6.0 (4.7±2.6)	1.0 1.5 2.0 (1.9±1.9)	1.5 2.0 3.5 (2.6±2.0)	0.0 2.0 4.0 (2.4±2.5)	0.0 1.0 3.5 (2.1±2.7)	0.5 1.5 4.0 (2.4±2.5)	0.0 3.0 5.8 (3.7±3.6)	2.8 4.2 6.0 (4.5±2.5)

$_a\ b\ _c$ represent the lower quartile a, the median b, and the upper quartile c for continuous variables. $x \pm s$ represents $\bar{X} \pm 1$ SD. N: number of samples available at each age group, it being possible to have more of one observation per subject.
