# Supplementary material for: Early Prediction of Alzheimer’s Disease Using Null Longitudinal Model-Based Classifiers
Source: PLoS One. 2017 Jan 3;12(1):e0168011. doi: 10.1371/journal.pone.0168011 (PMC5207395; doi:10.1371/journal.pone.0168011)
Supplement: S4 Table — (PDF) [file pone.0168011.s011.pdf]

S4 Table. Performances of classification for current diagnostic.

Table 4. Performances of classification for current diagnostic.

	Experiment	Features	ACC	SEN	SPE
Females	AD vs HC	F_1	91.7	92.8	90.0
		F_2	94.1	95.2	92.5
	MCI vs HC	F_1	79.5	92.9	52.9
		F_2	85.7	93.6	70.0
	AD vs MCI	F_1	66.8	57.3	74.3
		F_2	81.6	80.9	82.1
Males	AD vs HC	F_1	87.1	89.0	84.2
		F_2	94.1	97.7	88.4
	MCI vs HC	F_1	75.5	97.3	10.0
		F_2	82.5	93.5	49.7
	AD vs MCI	F_1	72.3	68.4	75.7
		F_2	73.8	64.7	81.3

F_1 is the features set integrated with the MRI-based biomarkers and age; F_2 is integrated with the MRI-based biomarkers, age, MMSE (Mini-Mental Examination Score) and CDRGLOBAL (Clinical dementia rating global scale).
