# Supplementary material for: Early Prediction of Alzheimer’s Disease Using Null Longitudinal Model-Based Classifiers
Source: PLoS One. 2017 Jan 3;12(1):e0168011. doi: 10.1371/journal.pone.0168011 (PMC5207395; doi:10.1371/journal.pone.0168011)
Supplement: S5 Table — (PDF) [file pone.0168011.s012.pdf]

S5 Table. Performances of classification for last known diagnostic.

Table 5. Performances of classification for last known diagnostic.

	Experiment	Features	ACC	SEN	SPE
Females	AD vs HC	F_1	86.6	90.7	77.7
		F_2	91.7	92.9	89.2
	MCI vs HC	F_1	70.0	70.0	70.0
		F_2	77.1	75.3	79.2
	AD vs MCI	F_1	69.8	86.5	45.0
		F_2	72.5	87.4	50.4
Males	AD vs HC	F_1	85.6	94.8	64.4
		F_2	90.0	96.8	74.4
	MCI vs HC	F_1	62.9	83.5	29.4
		F_2	77.6	85.0	65.6
	AD vs MCI	F_1	65.4	85.0	54.0
		F_2	69.0	85.1	5.9

F_1 is the features set integrated with the MRI-based biomarkers and age; F_2 is integrated with the MRI-based biomarkers, age, MMSE (Mini-Mental Examination Score) and CDRGLOBAL (Clinical dementia rating global scale).
